# Noise correlations in neural ensemble activity limit the accuracy of hippocampal spatial representations

**DOI:** 10.1038/s41467-022-31254-y

**Published:** 2022-07-25

**Authors:** Omer Hazon, Victor H. Minces, David P. Tomàs, Surya Ganguli, Mark J. Schnitzer, Pablo E. Jercog

**Affiliations:** 1 Stanford University, Stanford, CA USA; 2 University California San Diego, San Diego, CA USA; 3 Institut d’Investigacions Biomèdiques August Pi i Sunyer (IDIBAPS), Barcelona, Spain

**Keywords:** Hippocampus, Spatial memory

## Abstract

Neurons in the CA1 area of the mouse hippocampus encode the position of the animal in an environment. However, given the variability in individual neurons responses, the accuracy of this code is still poorly understood. It was proposed that downstream areas could achieve high spatial accuracy by integrating the activity of thousands of neurons, but theoretical studies point to shared fluctuations in the firing rate as a potential limitation. Using high-throughput calcium imaging in freely moving mice, we demonstrated the limiting factors in the accuracy of the CA1 spatial code. We found that noise correlations in the hippocampus bound the estimation error of spatial coding to ~10 cm (the size of a mouse). Maximal accuracy was obtained using approximately [300–1400] neurons, depending on the animal. These findings reveal intrinsic limits in the brain’s representations of space and suggest that single neurons downstream of the hippocampus can extract maximal spatial information from several hundred inputs.

## Abstract (web summary)

CA1 neurons encode an animal's position within the environment. Here the authors identified in hippocampal neuronal activity a detrimental type of noise that limits accuracy of spatial position, relative to body size.

## Introduction

The hippocampus encodes the spatial position of a subject in an environment^1^. This code likely originates from the combination of many sensory inputs and internal representations related to physical variables that contribute to position estimation^2–8^. Although previous studies have investigated the accuracy of decoding spatial position from neuronal activity, how that accuracy depends on population size has not been well-characterized experimentally. Because of the hippocampus’ prominent role in memory and spatial navigation, it is crucial to understand what bounds its accuracy. Thanks to modern techniques that allow us to record from a small but significant portion of the hippocampus, we could estimate, as we have in this work, the number of neurons necessary for encoding an animal’s position and the precision limits of that encoding.

Neurons in the CA1 area of the hippocampus modulate their firing rates as the animal visits different locations, yet their responses vary across visits^9^. This variability presents a limitation to any decoding mechanism built upon reading out neuronal responses to a set of stimuli. There are several strategies that the brain could use to cope with the effect of response variability. Experimental evidence suggests that one way to increase spatial coding accuracy is by the overrepresentation of place fields near locations related to task goals and salient sensory cues^10–17^. Previous influential work suggested that spatial accuracy increases with the size of the ensemble of neurons from which spatial information is decoded^18–22^.

A crucial factor in a network’s encoding capacity is what is called noise correlation. Each time an animal receives a given sensory input, for example, associated with a spatial location, a given neuron will respond with a variable number of spikes. The neuron’s responses can be characterized as an expected response (the mean over many presentations of that sensory input) plus a noise component that is different in each presentation. The noise components of two neurons might be correlated, meaning that over presentations of the same stimulus, the neurons will both respond with more spikes, or they will both respond with fewer spikes than expected. This correlation of noise components is called noise correlation. There is a long history of theoretical work indicating that noise correlations can greatly influence the capacity of a neural network to encode the world (for reviews, see^23,24^). If noise is not correlated, the response variability from different neurons can be averaged out, and a downstream reader reads the population’s expected response accurately. Conversely, positive noise correlations could distort the population response in a way that cannot be averaged out, leading to a deterioration of the encoding capacity. Noise correlation can be quantified as the Pearson correlation of a pair of neurons’ spike counts during the repeated presentation of the same stimulus. Several studies have identified noise correlation experimentally, for example^25–28^, among others. Noise correlations are a small portion of the neurons’ pairwise correlations, and their values are usually between 0.01 and 0.2^29^. The effect of noise correlation on the navigation system has been previously studied^30–32^. Yet their effect on spatial accuracy at large population sizes has remained unexplored until now.

The first theoretical studies that investigated the effect of noise correlations considered neurons with identical receptive field shapes^33–36^ and limited-range correlations (i.e., correlations that are strongest between pairs of neurons with similar preferred stimuli). Despite their great contribution, these models did not capture real neuronal response characteristics. When the models included neurons with receptive fields with heterogeneity in their shapes, the information contained in large ensembles was not limited by the presence of noise correlations^37,38^. This result has not been confirmed experimentally. The relation between receptive field shapes and the effect of noise correlation is still under investigation. In addition, ^39^ and ^40^ demonstrated that quantifying the effect of noise correlation on limiting information for large populations experimentally requires simultaneous recording from large ensembles of neurons.

Recent results in macaque prefrontal^41^ and mouse visual cortex^42,43^ have measured how information for large ensemble sizes is limited due to the presence of noise correlations. Those results pertain to sensory stimulus encoding, but very scarce data and theoretical predictions exist regarding high-order representations, such as the representation of space. It is important to note that brain areas encoding high-order representations, like the hippocampus, are formed by neuronal networks with various connectivity patterns (e.g., feed-forward, recurrent, or a mixture)^44^. These connectivity patterns allow hippocampal receptive fields, called place fields (PFs), to have unique properties, including attractor dynamics^45^, a lack of topography^46,47^, and global remapping^48^, among others. These unique properties yield further motivation for understanding how noise correlations affect coding in the hippocampus.

To assess how the hippocampal spatial representation is affected by noise correlations, we performed neuronal calcium imaging in the CA1 region of freely moving mice as they ran back and forth on a linear track to obtain a reward. We analyzed the accuracy of neural population representations of space in ensembles of up to ~500 simultaneously recorded neurons. Our analyses showed that noise correlations in the hippocampus were of the type that places an upper bound on the ensemble’s coding accuracy for ensembles larger than ~[300–1400] neurons. The results presented in this article were based on neuronal populations that consist of roughly 0.1% of the total population of the CA1 region. Still, the population sizes used here are larger than previous studies and sufficiently large to see the effect of noise correlation on information encoded by the neuronal population. Furthermore, we found that mice with more heterogeneous PF slopes displayed a smaller effect of noise correlation on limiting information at large ensemble sizes. In this work, we demonstrated the existence of information-limiting noise correlations in the hippocampal spatially-tuned neuronal activity and show how the heterogeneity of the population of neurons’ PFs is related to the information encoded by the neuronal ensembles.

## Results

**Fig. 1:**
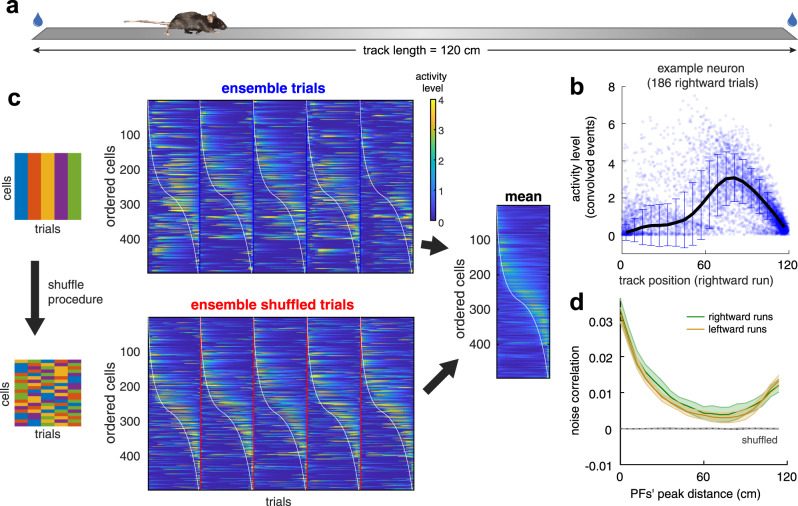
Noise correlation in the neuronal activity of the mouse CA1 area. **a** Mice ran back and forth along a linear track of length *L* = 120 cm and collected water rewards at each end of the track. **b** Example neuron’s activity level for different spatial locations. Activity level based on normalized amplitudes of detected events convolved with an alpha function (see Methods) for each visit to each spatial location (dots) relative to mean values across visits (continuous line). Error bars are the 95% confidence intervals over 186 trials. **c** **Left:** Schematic of the shuffling procedure, where we mixed neurons’ responses (columns) across different trials (rows) for the entire track. **Center:** Stack of maps of individual cells’ trial-by-trial PFs along the track for cells monitored together within a single imaging session, ordered by the maximum activity level for a given rightward running direction (for one session from animal 2022). Individual trials show response fluctuations relative to mean responses (Right) (the white line generated for visualization purposes was obtained from the leftmost end of PF’s widths (smoothed across neighboring neurons/horizontal lines) computed on the distribution of mean responses (Right)). **Right:** The mean response over trials for all recorded cells was unaffected by the shuffling procedure. **d** Noise correlations between neurons as a function of distance between PF peaks (3,941,140 pairs from 110 sessions from 12 mice). The solid lines and shaded areas represent means and 95% confidence intervals, respectively. The shuffling procedure drastically reduced noise correlations in the shuffled data. Source data is provided as a Source Data file.

To examine the statistical structure of hippocampal representations of space, we tracked the concurrent calcium dynamics of hundreds of CA1 pyramidal neurons in 12 mice (151-497 neurons per session, median 235; a total of 27,898 neurons over 110 sessions) that ran back and forth along a 120-cm-long linear track (Fig. 1a, see Methods). Because our study required the greatest possible statistical power, we used a linear track to increase the number of visits to each spatial location. We quantified CA1 neuron's calcium activity responses while the animals ran on the track. We detected spike events with our algorithm (see Methods and Suppl. Fig. 1) and found similar values to previous reports for PF shapes and spatial distributions (Suppl. Fig. 1 and Suppl. Table 1). PF characteristics were similar to those obtained with electrophysiological methods and other animal species (see Suppl. Table 1, Suppl. Fig. 1). In this study, we did not select cells based on the amount of spatial tuning, since cells that do not satisfy the traditional definition of “place-cells” can also contribute to the neuronal ensemble’s spatial information^49^. A position-responsive neuron varies its responses between individual runs (i.e., trials) through the track, relative to the mean response (Fig. 1b). Neuronal response fluctuations were, in general, large for different visits to each spatial bin (response variance = 0.7263 + /− 0.6117, variance between bins / variance within bins = single-cell signal-to-noise ratio = 0.41 + /− 0.34, pooled from 27,898 neurons).

When response variations were correlated among neurons with similar PFs, the estimation of mouse position deviated from the actual position. To illustrate this phenomenon, we first artificially eliminated noise correlations by randomly “shuffling” the neuronal activity from different trials in the session, independently for each neuron, and then compared the result with the neuronal responses from the original data (Fig. 1c, Suppl. Fig. 4, Methods). The shuffling procedure eliminated noise correlations for the neuronal ensemble while preserving the individual neuron's mean responses (Fig. 1c-right). Then, to show how noise correlations vary between neurons, we plotted the Pearson correlations of neuron pairs’ activities, arranged by relative distances between their PF peaks (Fig. 1d). In the hippocampus, we found that noise correlations’ dependence on PF distance was similar to previously measured values in other brain regions^25,27,28,50,51^. Finally, to test if the correlations were generated through contamination from nearby cells, we confirmed that pairwise correlations were independent of the physical distance of the neurons within the recording field of view, as previously demonstrated^47^.

**Fig. 2:**
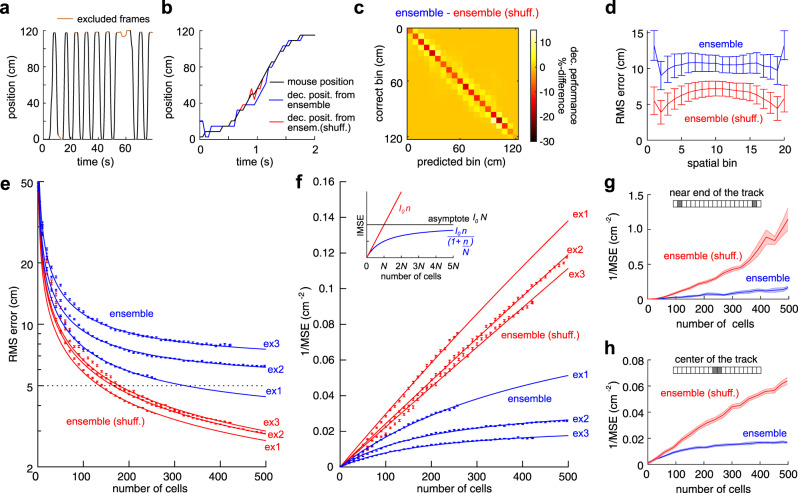
Noise correlation limits the spatial information encoded in the neural ensemble. **a** Position during running behavior (black). Each whole run through the track is considered a separate trial. **b** Example of one trial showing the actual (black) and decoded locations of the mouse using ensemble data (blue) and trial-shuffled noise correlation-free data (red). **c** Percent difference between the decoders’ confusion matrices (unmodified data minus shuffled data) for rightward runs. For all confusion matrices, see Suppl. Figure 4. **d** The root mean squared (RMS) error between predicted and recorded positions of the mouse, for each spatial bin, for unmodified data (blue) and noise correlation-free data (red), averaged over all 110 sessions. Error bars are the 95% confidence intervals of mean values. **e** RMS error from a neural decoder using unmodified (blue) and noise correlation-free (red) data as a function of the number of cells. Each data point denotes an average of over 80 randomly chosen subsets of cells. The data shown are from three different sessions with three individual mice (all sessions are shown in Suppl. Fig. 7). Solid lines follow the parametric fits in **f**. Error bars represent SEM over the random subsets of neurons trained with half of the trials and tested with the other half. **f** Inverse mean squared error (IMSE) as a function of the number of cells included in the decoder. Solid lines show parametric fits to the function shown in the inset. Error bars shown are based on the same data as in **e**. **Inset**: Graphical representation of the IMSE(n) = I_0_n/(1 + n/N) function used to fit the asymptotic behavior of the data. The parameter I_0_ represents the linear slope at a small ensemble size. N is the ensemble size where the IMSE equals half the asymptotic value. The product of the two, I_0_N, is the asymptotic value of the IMSE (Suppl. Fig. 7). **g** IMSE computed for one session with animal 2022 for the two bins closest to the end of the track (ends excluded because of behavioral variability). The shaded area represents the 95% confidence intervals of mean values of the IMSE for the valid trials in the displayed spatial bins. **h** IMSE computed for one session with animal 2022 for the bins in the center of the track. The shaded area is as in **g**. Source data is provided as a Source Data file.

To determine the impact of noise correlations on the accuracy of spatial information, we used decoders based on neuronal population activity, which we constructed using sets of linear support vector machines (SVMs) to estimate the mouse’s position and direction of motion from the neural activity (see Methods). To quantify how noise correlations affected the accuracy of spatial representations, we decoded the animal's position using the simultaneously recorded neuronal activity in 20 equal size spatial bins, with (i.e., “ensemble”) and without (i.e. “ensemble shuffled”) the presence of noise correlations (Fig. 2a, b). The misclassification error for ensemble activity data was worse than the case of ensemble activity without noise correlations. This effect was consistent across all spatial bins (Fig. 2c & Suppl. Fig. 5). The misclassification errors were typical of the order of one but could reach up to three spatial bins. Furthermore, the root mean square (RMS) error between predicted and recorded mouse positions showed that the effect of noise correlations was detrimental for position estimation across all spatial bins (ensemble: 9.38 + /−4.06 cm, ensemble shuffled: 5.15 + /−2.40 cm (median + /− IQR, *n* = 110 sessions); Fig. 2d, Suppl. Fig. 6).

To study the effect of noise correlations on spatial accuracy when ensembles grow in size, we decoded spatial location using simultaneously recorded neural ensembles. The decoding error approached an asymptotic minimum value for ensembles with large numbers of neurons. In contrast, the error decreased without apparent bound for the shuffled data (Fig. 2e). It is relevant to mention that the decoding errors were larger than the animal position tracking error (0.28 cm with 95% confidence intervals of [0.12−1.0 cm] across sessions; see Methods). To quantify the accuracy of a linear decoder, the inverse of the mean squared error (IMSE) can be used because of its relationship with linear Fisher information. Intuitively, the higher the Fisher information, the smaller the variance of the decoder’s estimates. Linear Fisher information can be mathematically represented as ${{{{{\bf{I}}}}}}\,{{=}}\,{{{{{{\bf{f}}}}}}}^{{{{\prime} }}{{{{{\bf{T}}}}}}}{{{{{{\boldsymbol{\Sigma }}}}}}}^{{{-}}{{{{{\bf{1}}}}}}}{{{{{\bf{f}}}}}}^{{{\prime} }}$, where ${{{{{\bf{f}}}}}}^{{{\prime} }}$ is the derivative of the population-tuning curve for the stimulus variable and ${{{{{\boldsymbol{\Sigma }}}}}}$ is the response covariance matrix. As expected from Fig. 2e for unmodified data, the IMSE appears bounded (Fig. 2f). On the other hand, no bound was present for shuffled data (Fig. 2f, (Suppl. Fig. 7a)), at least within the neuronal population size ranges we recorded. This result demonstrated the significant effect of noise correlations on limiting the accuracy of the spatial representation.

To estimate the ensemble size at which spatial information saturates, we fitted individual IMSE curves from different sessions to a function of the form ${{{{{{\rm{I}}}}}}}_{0}{{{{{\rm{n}}}}}}/(1+{{{{{\rm{n}}}}}}/{{{{{\rm{N}}}}}})$ that monotonically increases until reaching a plateau at ${{{{{{\rm{I}}}}}}}_{0}{{{{{\rm{N}}}}}}$ (Fig. 2f (inset), form^39^). In this function, *n* is the number of neurons in the ensemble, and ${{{{{{\rm{I}}}}}}}_{0}$ and N are fitting parameters. To quantify the number of neurons at which spatial accuracy starts to saturate in the IMSE curves (Fig. 2f), we chose the abscissa value corresponding to the point where the slope was 95% of the maximal asymptotic slope in the fitted function (see Methods). Beyond this point, the accuracy gained by increasing the number of neurons in the ensemble would be negligible. We validated our method for finding the saturation point; we created artificial data and compared the procedure with populations of 500 and 2000 neurons and found no significant difference in the saturation parameter estimates ${{{{{\rm{N}}}}}}$ (Suppl. Fig. 9). We found that the ensemble approached saturation in spatial accuracy with 346–1402 neurons (see Methods). These values were small if we compare them with the estimated pyramidal cell population size in mouse dorsal CA1 (e.g., 447,500 by^52^, and 165,742 by^53^). The asymptotic IMSE value (${{{{{{\rm{I}}}}}}}_{0}{{{{{\rm{N}}}}}}$) was significantly larger in the trial-shuffled data than in the ensemble data for all animals (Suppl. Fig. 7b–d) due to the elimination of noise correlations.

**Fig. 3:**
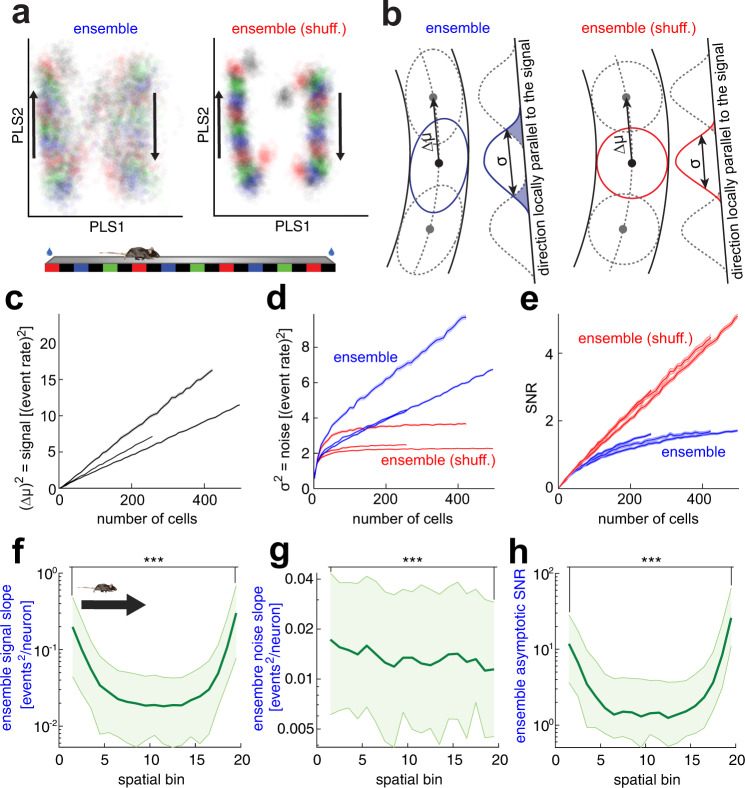
Signal-to-noise ratio as a direct observable to measure the effect of noise correlations on the neuronal representation of the linear track. **a** Projection of the first two dimensions identified by partial least squares (PLS) regression on position and direction of motion for each of the 40 bins from a single session with animal 2022. Same PLS components (from ensemble data) in both panels. Samples for each bin have different colors for visualization purposes. Each point on the plot represents the neuronal ensemble’s state within an individual trial and is color-coded according to the mouse’s spatial position (Bottom). The two leftmost clouds in each panel represent rightward runs, and the other two clouds leftward runs. **b** The schematic introduces Δμ as the distance vector between mean responses of adjacent spatial bins and *σ* as the neuronal activity standard deviation along Δμ. **c** Squared spatial signal, defined as the median squared distance between mean neuronal responses of adjacent spatial bins. The shaded regions are the 95% confidence intervals over 80 random subsets of cells from a single session with example animal 2022 as in **a**. The shaded areas are small, and most points are under the line representing the mean value. The plot shows the same examples as in Fig. 2d, e. **d** Unmodified data (blue) noise variance along the spatial position encoding direction. The fluctuations in the direction of spatial encoding are shared between neurons and cannot be eliminated by neuronal population activity averaging. Each plotted value is the median over spatial bins—shaded regions as in **c**. **e** SNR (signal in **c** divided by noise in **d**) as a function of ensemble size. **f** Asymptotic signal slope for different spatial bins on rightward runs. Signal slope decreases significantly between the beginning and end of runs (*p* = 2 × 10^−18^, one-tailed Wilcoxon signed-rank test, *n* = 110 sessions). Shaded regions are the 95% confidence interval over trials from one session with example animal 2022 (same as **a**). **g** Asymptotic noise slope displayed monotonic and significant decreases over the run (*p* = 8 × 10^−10^, one-tailed Wilcoxon sign rank test, pooling left and right runs, *n* = 110 sessions). Shaded regions as in **f**. **h** The asymptotic SNR (signal-slope / noise-slope) was significantly higher at the end of the run than at its initiation (*p* = 2 × 10^−29^, one-tailed Wilcoxon sign rank test, *n* = 110 sessions). Shaded regions as in **f**. Source data is provided as a Source Data file.

Up to this point, we have demonstrated that spatial accuracy in the hippocampus is subject to the effect of noise correlations on limiting information, but can we say something about the characteristics of the signal and noise that could explain this phenomenon? With the sole purpose of visualizing the relationship between signal and noise in hippocampal neuronal population activity, we generated a dimensionally-reduced projection of the population activity using partial least squares (PLS) regression. PLS is well suited for situations in which the number of trials is smaller than the number of neurons (see Methods). To visualize the effect of noise correlations, we compared response variability over different trials, with unmodified and shuffled data (Fig. 3a). The response variability was reduced for data without noise correlations (i.e., shuffled), and adjacent spatial bin representations appeared to be more discriminable (Fig. 3a, b). Noise correlations were present in the response variability in both PLS directions, but we focused on how variability affects the most susceptible direction for discrimination: the signal direction^39^.

Signal direction corresponds to the direction in which the population activity changes during changes in stimulus (i.e.,${{{{{\bf{f}}}}}}^{{{\prime} }}$). We approximated this direction by using the direction of the vector that connects the mean response between two adjacent spatial bins ($\Delta \vec{{{{{{\rm{\mu }}}}}}}$ in Fig. 3b). We computed the signal, noise and signal-to-noise ratio (SNR) between sets of two adjacent spatial bins within the 1-D projection defined by their signal direction. This is similar to quantifying the discriminability between two adjacent bins’ responses in the 1-D manifold plotted in Fig. 3a, b. The signal and noise components, in the signal direction, changed with the number of neurons included in the ensemble (Fig. 3c, d). In the unmodified data, the signal divided by noise (SNR, Fig. 3e) for large ensemble sizes reached an asymptotic maximum value in agreement with the IMSE in Fig. 2e. Since we had access to the linear growth rates of the signal and noise independently, we inferred the asymptotic SNR value for each session as the signal slope divided by the noise slope. Across all mice, the asymptotic SNR values were consistently lower in the unmodified data than in the shuffled data (Suppl. Fig. 10). Our definition of asymptotic SNR shows a linear behavior that allows us to estimate the effect of noise correlations on spatial accuracy even for smaller ensemble sizes of approximately 100 neurons; an ensemble size that would not show clear signs of saturating decoding accuracy otherwise (Fig. 2f). The SNR varies across the entire linear track due to differences in mouse behavior, but despite these differences, the behavior of the asymptotic SNR is similar for different spatial locations. Figures 2d, g, h illustrate the differences in decoding errors for different spatial bins. Thus, the asymptotic SNR is also different for each spatial bin but remains bounded for the entire track (Fig. 3f–h), regardless of the degree of PF density along the track (Suppl. Fig. 2b).

**Fig. 4:**
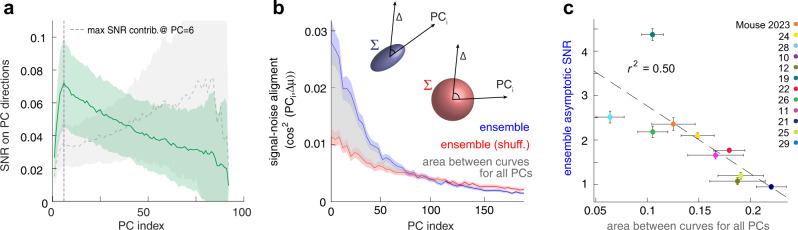
Spatial information is distributed across many dimensions. **a** The SNR along each noise covariance matrix eigenvector. SNR computation using the training set (dotted gray) or the testing set (solid green). Median and the 95% confidence interval (shaded area) over spatial bins, averaged over 100 different train (gray) and test (green) sets and aggregated over 73 sessions with 10 mice. The first dimensions increasingly accumulate contribution to SNR, peaking at PC_6_ (purple dashed line). The accumulated contribution up to this point is a small percentage of the area under the curve as the PC indices grow to large numbers. **b** cos^2^ between the signal direction Δμ and the noise mode direction PC_i_ for ensemble data (blue) and ensemble shuffled data (red). Shaded area is the 95% confidence interval based on the same testing set as in **a**. The gray area between red and bluecurves is an empirical estimate of the overlap between signal and noise due to noise correlations. Gray area is computed using all available PC directions per session, which were limited by data size. **c** The total cos^2^ overlap between signal direction Δμ and each noise PC direction in the shuffled data, subtracted from the corresponding quantity in the unmodified data, correlates with the asymptotic SNR from Supp.Fig. 11c. Increase in signal-noise overlap due to noise correlations predicts the limit of spatial information (Pearson’s corr. = 0.81, r^2^ = 0.50, *p* < 0.05, *n* = 73 sessions with 10 mice. Each dot represents the average over sessions for each animal, error bars are the 95% confidence intervals over each animal’s sessions). Noise structure explains how noise correlation limits hippocampal spatial information. Source data is provided as a Source Data file.

Two important questions could be investigated using the results and data presented above: 1) the potential dimensionality of the spatial neuronal code in the hippocampus and 2) how the distribution of noise components in the high-dimensional neuronal space explains the saturation of information for large neuronal ensembles. Dimensionality reduction has been the basis of many data analysis developments in systems neuroscience in the last years (for review see:^54^). These methods estimate the multi-dimensional structure of neural population activity based on correlations among simultaneously recorded neurons. In hippocampus research, these methodologies have recently uncovered that low-dimensional representations (3–4 dimensions) are sufficient to understand a large fraction of brain activity^55,56^ when animals move in environments with simple geometries, as is the case in our experiment. It has been proposed that, for the number of neurons and trials in our experiment, a good estimate of the dimensionality of the neuronal population activity could be obtained by computing the number of noise components (i.e., the eigenvectors of the noise covariance matrix, PC) that accounts for most of the shared variance of the neurons’ responses^57^. Intuitively, if the neuronal activity is low-dimensional, most neurons will co-activate because they share information and therefore have a single dominant eigenvector of the covariance matrix. On the contrary, if each neuron carries unique information with low shared fluctuations, the number of eigenvectors with significant amplitude will be high. To test this, we first obtained the noise correlation covariance matrix eigenvectors. The distribution of noise components can be visualized by picturing the principal axes of an ellipsoid in a high-dimensional space that contains all the noise correlations among all simultaneously recorded neurons. We computed the SNR within each principal noise component to determine how much information each component carries (Fig. 4a). We found that each noise component’s contribution to the SNR increased until it reached its maximum at the sixth largest component (for individual animal's results, see Suppl. Fig. 12–14). The first six components only accounted for a small proportion of the neuronal ensemble’s total information. This proportion is most likely to be even smaller if one considers the contributions from higher noise modes (the continuation of the green curve, converging to a residual SNR close to zero, Fig. 4a) that we could not accurately compute because the number of trials was smaller than the number of recorded neurons. Therefore, to recover most of the spatial information it is necessary to observe a large number of dimensions encoded in the neuronal activity (much larger than six). The distribution of SNR components shows that information in the hippocampal spatial representation is distributed broadly over many dimensions, in contrast with previous reports in neocortical areas, where a high percentage of the information was concentrated in the first few components^41,42^. Our result suggested that the way information is encoded and transmitted in the hippocampus differs from the neocortex.

Is it sufficient to find noise correlations in cell pair recordings (as shown in Fig. 1d) to claim that information will saturate with ensemble size? The answer to this question was proposed by^39^ indicating that only if noise correlation distribution has a component parallel to the signal direction can it limit information for large ensemble sizes. Intuitively, this is when the noise ellipsoid in the high dimensional space containing the neuronal population noise has a non-zero projection onto the signal subspace direction. Meaning that the larger and the more principal noise components align with signal direction, the faster the spatial information saturates as the ensemble size grows. To experimentally test^39^ prediction in the hippocampus, we quantified the alignment of noise with signal direction using the squared cosines of the angles between each noise covariance matrix’s ${{{{{\rm{P}}}}}}{{{{{{\rm{C}}}}}}}_{i}$ and signal direction $\Delta \vec{{{{{{\rm{\mu }}}}}}}$ (i.e., ${{\cos }}^{2}({{{{{\rm{P}}}}}}{{{{{{\rm{C}}}}}}}_{i},\triangle \mu )$, Fig. 4b). Since ${{\cos }}^{2}({{{{{\rm{P}}}}}}{{{{{{\rm{C}}}}}}}_{i},\triangle \mu )$ only describes the overlap between one noise component and signal direction $\Delta \vec{{{{{{\rm{\mu }}}}}}}$, we used the overlaps over all PCs to compute the overlap between noise and signal direction (Fig. 4b). We were particularly interested in to what extent noise correlations increased this total overlap. Therefore, we used the change in overlap between the unmodified and noise correlation-free data to quantify the degree to which noise correlations affected the alignment of noise with the signal direction (Fig. 4b, gray area). We found that the average change in total overlap between noise and signal direction was inversely correlated with the asymptotic SNR slope (i.e., Fig. 3e & Suppl. Fig. 10b) across animals (Fig. 4c, *r*^2^ = 0.50, *p* = 0.014), confirming the intuition. With this result, we have empirically demonstrated that the purely geometric property of the overlap between signal direction and the shape of the noise structure predicts the limit at which the ensemble information saturates.

So far, we have presented results describing phenomena that only exist in high-dimensional spaces where neuronal population activity resides, but their connection with individual neuron properties is not direct. Thus, how do our results relate to biological quantities, such as the shape of the PFs? Is it possible to describe how noise correlations limit spatial information based on single neuron properties? The question of how PF shape determines spatial decoding accuracy has been at the center of the discussion in the field of hippocampal research since the discovery of place-cells (^for examples see58,59^, among others). We tried to provide new insights on this matter, considering the effect of noise correlations. Previous theoretical studies demonstrated that the heterogeneity of the amplitudes and widths of the neurons’ receptive fields in a given ensemble mitigates or avoids information saturation as ensemble size increases in the presence of noise correlations^37,38^. Kanitscheider et al.,^40^ instead showed that, in the scenario of limited input information, the output information would be saturated by the presence of noise correlations for both homogeneous and heterogeneous receptive fields. To provide experimental evidence to support either of these two theoretical predictions regarding the effect of PF heterogeneity on decoding spatial accuracy for large ensembles, we once more exploited the variability among animals in terms of spatial encoding accuracy.

**Fig. 5:**
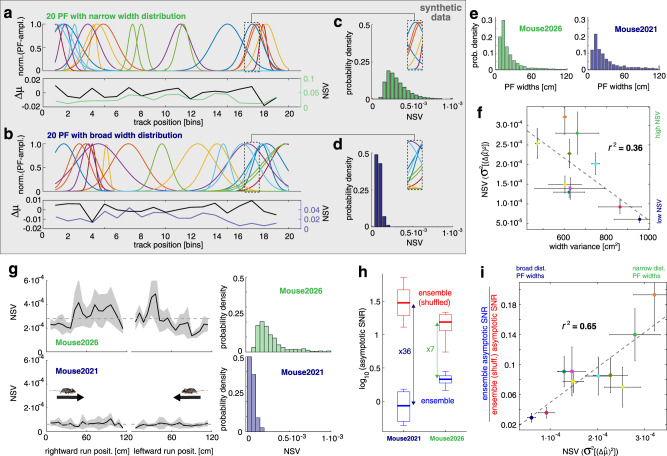
PF shape heterogeneity explains the effect size of noise correlations on decreasing spatial representation accuracy. **a** **Top:** Synthetic distribution of narrow PFs generated by 50 Gaussian curves with a uniform width distribution of min = 0.2 and max = 1.42. **Bottom:** Average over the distribution of synthetic PF slopes within each spatial bin (black), and the variance of the normalized PF slope distribution, called normalized signal variance $({{{{{\rm{NSV}}}}}}={\sigma }^{2}[{(\hat{\varDelta }{{{{{\rm{\mu }}}}}})}^{2}])$, between two spatial bins (red). **b** Same as in (a), but with a uniform width distribution of min = 0.8 and max = 2.0. The distributions were chosen from a parameter study to match our recorded data (Suppl. Fig. 16). **c** Distribution of NSV from each bin, generated from 1000 simulations of the PFs in **a**. **d** Distribution of NSV from each bin, generated from 1000 simulations of the PFs in **b**. The variance of the PFs determines the shape of the PF slope distribution (inset rectangles, top-right). **e** Width distributions for two example animals: 2026 (narrow distribution) and 2021 (broad distribution). **f** Correlation between animal’s PF width and NSV. The broader the width distribution, the narrower the NSV values per animal. Pearson’s corr. = −0.65, r^2^ = 0.36, *p*_val_ = 0.039. Each dot represents the average over sessions for each animal. Error bars are the 95% confidence intervals over sessions. **g** NSV for each bin from the linear track for one session. Two extreme example animals: high NSV (Top: Mouse2026), low NSV (Bottom: Mouse2021). The dashed line represents NSV averaged over bins. Black lines and shaded areas correspond to the mean and the 95% confidence intervals over sessions. NSV distribution for all bins, from all mouse sessions (Left). **h** Asymptotic SNR changes after eliminating noise correlations (shuffle). The effect is higher in animals with a small NSV. Each thick line represents the median over sessions for each animal. Boxes top and bottom edges represent the interquartile range (IQR). Whiskers minima is the (first quartile)-1.5*IQR and the maxima is the (third quartile) + 1.5*IQR. **i** Correlation between the asymptotic SNR ratio (i.e., ensemble with noise correlations over ensemble shuffled) and averaged NSV (Pearson’s corr. = 0.83, r^2^ = 0.65, p_val_ = 0.003). Each dot represents the average over sessions for each animal. Error bars are the 95% confidence intervals over sessions. Animals whose neuronal representation had a narrow PF width distribution were more affected by noise correlations. Source data is provided as a Source Data file.

We did not find a direct correlation between the variance of PF amplitudes or widths with the effect of noise correlations on information saturation. Instead, we found that the distribution of individual PF slopes could explain the effect of noise correlations on saturating spatial information for large ensembles. To provide some intuition about this finding, we first used synthetic data to describe, in a simplified manner, the population of PF shape heterogeneities displayed in our recordings. In the synthetic data, PFs have a Gaussian shape with different widths. Different width distributions lead to different distributions of PF slopes between adjacent spatial bins. For illustration, we plotted two distinct PF width distributions (narrow and broad, Fig. 5a, b). Using this simplified description, we can easily show how the distribution of PF slopes between two spatial bins relates to the distribution of PF widths, a more commonly used quantity. To complete the picture of our new variable, we now describe the distribution of slopes between consecutive bins (described by **f’** for the ensemble or $\Delta \vec{{{{{{\rm{\mu }}}}}}}$ for a single neuron). We computed the normalized signal between two spatial bins: ${\hat{\varDelta} \mu }=\Delta \vec{{{{{{\rm{\mu }}}}}}}/|\Delta \vec{{{{{{\rm{\mu }}}}}}}|$, where each element of this vector represents the slope between two consecutive bins for each neuron in the population. We then computed the variance of each of the squared elements of ${\hat{\varDelta} \mu }$. We called this new statistical quantity the normalized signal variance (NSV): ${VAR}[{({\hat{\varDelta}} \mu)}^{2}]$, representing the spread or variability in the sensitivity of each neuron's response for the position (see Methods). Although counterintuitive, the narrower the distribution of PF widths, the broader the distribution of PF slope, quantified by the NSV (Fig. 5c, d). This inverse relation depends on the size of the environment and the number of active neurons. We confirmed that this relation remains qualitatively the same for populations as large as 10 thousand neurons (Suppl. Fig. 17). To demonstrate that our simplified model of PFs is a good description of the biological data, we computed the distribution of PF width for two example animals (Fig. 5e). We could confirm the observation from synthetic data that there was an inverse relationship between the variance of PF width and NSV in the real data (Fig. 5f). Different animals presented different mean NSV distributions over the linear track (Fig. 5g, Suppl. Fig. 18). We found that, for each animal, the smaller the NSV the larger the ratio between asymptotic SNR of the unmodified data and the asymptotic SNR of the data without noise correlations (i.e., asymptotic SNR ratio) (Fig. 5h). Therefore NSV explained the noise correlations’ effect on limiting the spatial information (Fig. 5i, *r*^2^ = 0.65) over the distribution of the recorded animals. In addition, NSV described the relation between noise correlation and asymptotic SNR ratio better than other variables that parametrized PF shape (see Suppl. Table 2 for comparisons with other quantities). We confirmed that our result does not depend on the number of neurons active or trials performed in a given session (Suppl. Table 2 and Suppl. Fig. 19). This analysis concluded that the narrower the distribution of PF slopes between consecutive spatial bins, the larger the effect of noise correlations on limiting information at large ensemble sizes. In this way, we connected the effect of noise correlations on limiting information to individual neurons PF properties.

## Discussion

For many decades, the standard view in the fields of spatial memory and spatial navigation research has been that an increase in spatial accuracy could be achieved by increasing the neuronal ensemble size involved in the representation of a given environment^18,19,22^, having consequences in current investigations. However, theoretical and experimental work in neocortical areas has shown that, in the presence of noise correlations, the decoding accuracy cannot be unbounded; it must reach a maximum that is independent of the size of the neuronal population participating in the representation. We have experimentally demonstrated that noise correlations limit spatial representation’s asymptotic accuracy for large neuronal ensembles in the hippocampal network activity. Our result coincides with several observations in the neocortex^41–43^, but we have demonstrated these phenomena in the hippocampus. For this reason, we believe it is of great relevance for the field.

Noise correlations could be the manifestation of information that was not taken into account in our decoders (i.e., slow oscillations, phase-precession, speed, acceleration, heading-direction relative to landmarks, time of day, odors in the room, etc.,) (e.g.,^49,60^). It was recently shown that it is possible to decode information related to body movement from the visual cortex; however, this information is mainly perpendicular to the signal direction of the visual stimuli^61^, which, in principle, would not affect decoding accuracy. Stringer et al.,^61^ have shown thatvisual and body movement representations share only one dimension. Further analysis eliminating noise correlations should be performed on their data to estimate the effect of body movement on visual information for large ensemble sizes. The hippocampus has a different structure and connectivity properties than the visual cortex. Therefore the results of Stringer et al., might not generalize to the hippocampus.

As remarked upon by^62^, our division of neuronal activity into signal and noise is operationally defined based on the spatial position variable that we measured in the experiment. We cannot rule out that adding more observational variables to the analysis would better explain the neural responses quantified by the variance explained. To test the hypothesis that other variables could contribute to information-limiting noise correlations, an experimenter would need to proportionally add as many trials as new bins are added to discretize the extra encoded variables. In our data, this would be unfeasible because it would dilute the statistical power. Nevertheless, we encoded two observational variables: position and heading direction (rightward and leftward runs). We used a total of 40 spatial/directional bins in our analysis. In our case, this treatment of the data improved decoding accuracy.

Recent work has shown that eliminating noise correlations decreases decoding accuracy when the position is estimated from ensembles of ~350 neurons from the hippocampal area CA1 of mice freely exploring an open field^49^. The reported opposite effect relative to our findings is likely caused by their shuffling method not controlling for the direction of motion, in conjunction with shuffling the calcium event times before convolving the event traces with a transient function. These two factors degrade the benefit of the convolution to the decoding accuracy and are absent in our methodology. The shuffling procedure of Stefanini et al., is conceptually different from ours because our results are obtained on the premise of finding the absolute minimal error, which is relaxed in the case of mixing visits with different heading directions. Peyrache et al.,^31^ also claimed that noise correlations improve decoding accuracy in areas connected to the CA1 hippocampal region. In this case, the authors simultaneously recorded from a modest number of neurons (2–12 cells), making it difficult to derive a conclusion due to the poor coverage of the encoded variable. As we have found in our study in Figs. 3 and 4, a minimum of 100–150 neurons were necessary to describe the effects of noise correlations on asymptotic decoding accuracy.

We discovered that spatial information is distributed over a large number of dimensions, which makes the hippocampus different from previously reported neocortical areas. Hippocampal area CA1 seems to be a region where considering only the principal noise components with the largest SNR is insufficient to estimate the encoded information accurately. Because we found that information is encoded in a large number of dimensions, one could hypothesize that hippocampal neurons integrate inputs from a larger population of presynaptic cells than neocortical neurons do. Previous theoretical work^57^ proposed that networks should display high levels of clustering in subpopulations of neurons to concentrate the noise in the largest components. Instead, based on our experimental observations, the level of neuron clustering in the hippocampus might be low or negligible due to the broad SNR distribution over many components, giving a potential argument as to why the hippocampus shows a different  noise distribution than neocortical areas.

It has been shown that attention and learning can change the effect of noise correlations on information^26,63,64^. Despite the large variability in PF shape, we could determine the impact of noise correlations on spatial information between different animals by using a statistical quantity we termed the NSV. This provided experimental evidence showing how PF heterogeneity reduces encoding accuracy due to noise correlations. One natural question is whether PF heterogeneity can be modified in the medial temporal lobe circuit to increase the accuracy of representations, for example, during learning. The answer to this question will be the subject of further analyses and novel hypotheses. Currently, hippocampal research uses spatial information^58^ to describe spatial accuracy, which is a quantity that increases the narrower the peak (or multiple peaks) of the PFs become. Some experimental evidence from animals accumulating experience running on linear tracks reported broadening PFs^65^, while others reported sharpening^66^. Instead, we found that the effect of noise correlations in changing the information encoded in the hippocampus was reduced if the PF NSV was more heterogeneous, rather than if the neurons’ PFs became narrower or broader. The differences between our and previous results are due to differences in the study approach for hippocampal spatial representations: the single neuron versus neuronal population representation. For the traditional single neuron and deterministic doctrine, information is quantified based on the PF’s mean response . This view ignores the effect of the PF’s response being highly variable between different visits to the same spatial location and how this interplays with the redundant information from neurons with overlapping PFs. Our result should encourage the analysis of spatial accuracy taking into account the effect of trial-by-trial variability using tools such as decoders. It should also inspire new experiments to describe in detail how PF heterogeneity evolves during learning and forgetting.

The methods that we have developed to observe the asymptotic behavior of spatial accuracy at large ensemble sizes using the SNR between representations of two spatial bins enable the measurement of the effect of noise correlations even on ensembles of modest sizes (~100 neurons). Thus, this methodology now provides a way to observe changes in spatial accuracy during learning and forgetting. Furthermore, our method could be used during experimental manipulations involving optogenetic and pharmacological techniques, even when ensembles of neurons’ activities are collected via traditional methods like tetrode recordings.

What are the implications of having limited accuracy in the spatial representation encoded in the dorsal hippocampus? We observed that the asymptotic decoding error was, on average, 9.38 +/− 4.06 cm (median +/− IQR). Ten centimeters is approximately the size of a mouse’s body, and it is possible that, on average, the animals did not require higher precision to navigate in our task. This spatial error might not be an absolute lower bound but the manifestation of the accuracy required to solve a simple linear track task. We propose that, in more demanding spatial navigation tasks, the spatial error might decrease (as suggested by Supp. Fig. 8c), and perhaps PF heterogeneity might also change accordingly. On the other hand, in tasks such as free exploration or random foraging, the error can increase.

We have experimentally demonstrated the existence of information-limiting noise correlations in hippocampal neuronal activity, and we have provided experimental evidence of how the heterogeneity of neurons’ PFs can counteract the information-limiting effect of noise correlations. Furthermore, we proposed that PFheterogeneity can be modulated to increase the information encoded in the hippocampus’ neuronal activity in processes such as learning during spatial navigation, as suggested by our analysis in Suppl. Fig. 8c. Finally, we put forward the idea that, to better quantify how information increases during learning, it is necessary to consider the effect of noise correlations on neuronal population activity.

## Methods

### Animals

Male C57BL6/J mice (Jackson Labs; 14–16 weeks old) were provided food *ad libitum* and water restricted to 0.5 mL per day plus the water obtained during the task (0.5–1.5 mL). Before the experiment, mice were habituated to human handling twice a day for three days. All animal procedures were approved and executed following institutional guidelines (Stanford Administrative Panel on Laboratory Animal Care).

### Behavioral box

The arena was a linear corridor 5 cm wide and 100 cm long, with two, 10 cm deep, trapezoidal compartments at the two ends. Water spouts were located at the back end walls, leaving enough room for the animal to turn inside the compartments. The water was delivered by a solenoid valve driven by an Arduino that controlled the amount of water delivered and kept track of the alternating water availability. Water was released when the animal closed the electric circuit between a metal base and the dispenser tube.

### Behavioral task

The animals ran on a linear track to collect rewards (50 µL water drops) from the two water spouts located at both ends of the track (Fig. 1a). Mice performed more than 100 trials per session. The task was based on collecting rewards at the two ends of the track without repetition. The mice did not receive the reward if they returned to the same end. A five-day pre-training phase was used to teach the animals to run to alternate ends of the track to obtain rewards and reach many runs.

### Analysis of behavioral data

Each session’s output data consisted of a full video recorded from a camera above the arena and synchronized frame by frame with the mini-microscope’s recorded frames. Videos were recorded using IC-capture. Frames were triggered by the TTL signal from the mini-microscope DAC board output. The recording frequency was 20 frames per second. The tracking of the mouse’s position was performed using a MOG algorithm implemented in OpenCV. We discretized the animal's spatial position on the track into 20 bins of equal length, each approximately 6 cm long, for each running direction. This selection was based on minimizing the decoding error (Suppl. Figure 4) and maximizing the number of samples per bin to obtain higher statistical significance in analyzing noise structure. During each 20-minute session, the mice performed up to ~380 runs over the track in each direction, which we called trials.

### Estimate of position measurement noise

Noise in the estimates of the mouse’s positions from the overhead camera can, in principle, reduce the position decoder's performance and introduce noise correlations that limit information. However, we estimated that this noise source was minimal compared with our typical decoding errors on the scale of 10 cm and that it did not significantly influence our results.

To assess the degree of position measurement noise, we took each running trial and isolated the frames between spatial bins 7 and 13 in the middle of the track (center-track frames). Between these bins, the motion appears to be uniform in most trials. We sought to measure the spread of the residuals relative to a linear model of uniform motion. Our approach used half of the center-track frames from each trial to fit a linear model between time and position and then measure the residuals’ spread using the other half of the frames. We first collected the residuals from each trial, we then measured their collective standard deviation using a robust estimator, resulting in the estimated scale of the position measurement noise for a given session. The robust estimator was necessary since a minority of the trials exhibited non-uniform motion, inflating the residuals and creating outliers. We used the interquartile range (IQR) with a normalization factor to match the result with the standard deviation of a Gaussian distribution. On average, the scale of the jitter was 0.28 cm, with a range of 0.12–1.0 cm across 110 sessions. Therefore, the position measurement noise cannot explain the observed saturation in the spatial information of ensemble activity.

### Viral injection

Surgeries were performed when the mice were between 6-8 weeks of age. Only excitatory pyramidal neurons were labeled by injecting adeno-associated virus (AAV, serotype 2.5), driving expression of GCaMP6m via the CaMKIIα promoter. We injected 600 nL of the AAV (injection coordinates relative to bregma divided between three locations: mediolateral (ML) = 1.8, anterior-posterior (AP) = −1.5, dorsoventral (DV) = −1.6; ML = 1.4, AP = −2.2, DV = −1.55; ML = 2.1., AP = −2.9, DV = 1.8) via a borosilicate glass pipette with a ~50-μm-diameter tip using short pressure pulses applied with a picospritzer (Parker Hannifin).

### Mini-endoscope implantation

Thirty days after virus injection, a second surgery was performed to implant a mini-endoscope, which is a stainless steel guide tube (1.2 mm diameter) with a custom glass coverslip glued to one end (0.13 mm thick cover glass, Paul Marienfeld GmbH), which holds the GRIN lens to focus the image on the focal plane of the mini-microscope. We inserted the mini-endoscope with the position and the angle to cover the more extended flat area of the dorsal part of the hippocampal CA1 region (relative to bregma ML = 2.1 (+77° on the coronal plane), AP = −2.2, DV = −1.1(from dura)). To prevent increased intracranial pressure, all brain tissue inside the cylindrical volume that the mini-endoscope occupied was aspirated, removing up to the second set of fibers crossing over the CA1 area, coming from the entorhinal cortex. Each group of fibers was identified by its orientation ~60 degrees from the previous layer. Mice recovered after three days, but we waited up to 5–7 weeks for the tissue to return to its place after movement due to a neuroinflammatory process. At this point, the level of GCaMP6m expression was checked by bringing the mini-microscope to the GRIN lens on a head-fixed mouse and determining if neurons were activated when the animal ran on a wheel. Once the level of expression was constant, we then mounted the miniature microscope’s base plate (nVista HD, Inscopix Inc.) using acrylic cement and ultraviolet-light curable glue.

### Calcium recordings during behavior

We used a computer to control and store the nVista mini-microscope frames and send the trigger inputs to the camera that recorded the behavior. We then combined the calcium activity with animal behavior offline. First, the raw movies from the mini-microscope were downsampled before processing due to computer memory constraints. Next, we used the NoRMCorre piecewise linear registration algorithm^67^ to minimize the frame-to-frame displacements caused by the animal’s brain movement relative to the mini-microscope field of view. Next, $\Delta F/{F}_{0}$ was obtained by subtracting and dividing each pixel value at a given frame by its mean activity across the recording session. This operation was followed by applying the CELLMAX extraction algorithm^68^, which models how the movies arise from the underlying calcium signals and finds the most likely set of neurons in the movie by computing the maximum likelihood of this probabilistic generative model. By applying this algorithm on a temporally downsampled version of the $\Delta F/{F}_{0}$ movies, we obtained 600 to 1000 mask candidates for the neurons in a given session. Finally, these candidates were inspected in a semi-automated manner for calcium-like dynamics and neuron-like shapes, resulting in ~200–500 simultaneously recorded neurons per session.

### Event detection algorithm

To generate event traces from calcium $\Delta F/{F}_{0}$ traces, we performed the following steps:

1. For each trace, we performed wavelet denoising using the wdenoise function in MATLAB with the default options. Then, we subtracted the denoised trace from the original to recover the removed noise component. Wavelet denoising excels at removing Gaussian noise, such that the local optima in the denoised trace are much less likely to be spurious and more likely to correspond to calcium events.
2. We set the minimum event size to three times the standard deviation of the noise component.
3. We set the minimum event peak value to the first quartile of the denoised trace, plus the minimum event size.
4. We saved all of the local maxima in the denoised traces that were higher than the minimum event peak value. These were the putative event peaks.
5. We found the local minima preceding each aforementioned local maxima. Thus, these were the putative event baselines.
6. We found the frames between the baselines and peaks where the denoised trace surpassed the baseline value plus the minimum event size. These frames were the putative event onset.
7. We removed the putative events whose peak values were below the minimum event size higher than their baseline values.
8. We generated event traces containing zeros except at the event onset frames, where we set the values to the corresponding event peak values minus their baseline values.
9. We convolved each event trace with the function $\alpha \left(t\right)=\left(t/\tau \right){\exp }\left(1-t/\tau \right)$, where $\tau$ = 0.2 sec sampled at 20 Hz from *t* = 0.05 to 1.5 s. This convolution simulates a spike burst from each calcium event.
10. Finally, we divided each event trace by its own standard deviation.

We used this method because it does not require estimating a baseline level for each trace. In many of the neurons, the periodic running activity of the mice led to periodic and dense activity patterns that obscured the true baseline value. Instead of looking for peaks at a certain threshold above the baseline, our method estimates the noise level and searches for peaks of a relative magnitude that exceed the noise level by a specific factor.

### Data preprocessing for decoding mouse position

Out of 239 total sessions from 12 mice, in the analysis, we included only those sessions surpassing 150 identified neurons and 30 running trials in each direction of motion (110 sessions from 12 mice, the “working” sessions). In some analyses involving estimating the correlation matrix structure (Fig. 3g and the following figures), we opted to further restrict the selection of sessions to those with at least 200 neurons. We referred to the limited subset of sessions as the “large-data” sessions (73 sessions from 10 mice). Within each session, we defined a valid trial as a contiguous duration in which the speed of the mouse was always greater than four cm/s, and the mouse visited each spatial bin on the track. Each trial contained either leftward or rightward motion. We discretized the position variable into 20 bins and arranged the neuronal data into an activity tensor with three axes: neurons, spatial bins and trials (Suppl. Fig. 10). The (*n*,*k*,*t*) index of the tensor contained the mean event trace value for neuron *n*, during trial *t*, from all the frames where the mouse was in spatial bin *k*. Note that the choice to integrate all frames from a single visit into a spatial bin as the neuronal representation for that visit means that the effective integration time differed based on the average velocity during each visit. We chose this scheme to be consistent with the model of a spatially parametrized population-tuning curve that is also spatially discretized. The position decoder used either the activity tensor as-is (i.e., “ensemble”) or the tensor’s trial-shuffled version. To perform the trial shuffle, we randomly permuted the activity values of each neuron across trials but within the same spatial-directional bin (Suppl. Fig. 2). We randomly selected 50% of the trials to serve as held-out test data for decoding, while the other 50% served as a training set. In the cases where trial-shuffling was necessary, we shuffled both the training and testing sets separately. Note that shuffling only the training set but not the testing set is not equivalent to our approach and may produce qualitatively different results.

### Computational spatial position decoder

We used a multiclass ensemble of linear support vector machines (SVMs) to decode the spatial position from neural data. These decoders had the ability to account for the noise correlation structure during training^69^ and had access to all neurons regardless of their spatial coding properties. Stefanini et al.,^49^ demonstrated that cells that do not encode spatial information may still contribute to the population code. Such decoders can, in principle, perform either as well as or better than equivalent decoders that ignore the correlation structure, such as naïve Bayesian decoders^23^. Each class represented a combination of spatial bin and direction of motion, with 20 classes for each direction of motion, making a total of 40. There was one SVM binary classifier between each pair of classes. We used the decoder to predict each column’s class from the aforementioned neuronal activity tensor, in which a column represents the population activity within a spatial bin on a given trial. The ensemble of binary classifiers voted to decide on the decoder’s predicted class.

### The rationale for discretizing the position variable

Our analyses were based on the decoding accuracy of a classification model that predicts a discretized position variable. Here we explain the choice to discretize the position variable. The problem of predicting the mouse’s position and direction of motion based on neuronal activity naturally lends itself to a regression model. The neuronal activity values are numeric predictors and the position target variable is likewise numeric. Thus, a model that jointly uses regression and classification to predict the position and the direction of motion would have sufficed to characterize the accuracy with which hippocampal neuronal activity encodes these variables. However, the goal of our analysis was to compare the encoding accuracy of the unmodified data to that of trial-shuffled data. To trial-shuffle continuous position data, one must equivocate visits to nearby yet non-identical positions, treating them as close enough to perform the shuffle between them. The simplest way to carry out that equivocation is by discretizing the position variable. All frames with positions within the same spatial bin and the same direction of motion are eligible to have their neuronal activities shuffled with other frames in that bin but not with those in other bins. Since the problem now becomes to predict spatial-directional bins based on neuronal activity, we opted to use a linear SVM-based classifier. It may be possible to define a trial-shuffle that keeps the position variable continuous and chooses to shuffle a pair of frames based on a probability that decays as a function of the distance between the frames’ positions. However, such an approach would be more challenging to interpret, and its results would be harder to compare to earlier work that shuffled position-labeled neural data via discretization^49^.

### Determining where spatial accuracy approaches saturation

The IMSE of decoding spatial position as a function of ensemble size was estimated by computing the mean IMSE using randomly sampled subsets of neurons of a particular size for a range of ensemble sizes. Each subset of neurons was drawn independently of the others so that they were not necessarily disjointed. Error bars were computed based on the SEM over these subsets. When decoding from shuffled data, we applied the trial shuffle separately to each random subset, and we then fit the IMSE curves as a function of ensemble size to the function ${{{{{\rm{IMSE}}}}}}(n)\,=\,{{{{{{\rm{I}}}}}}}_{0}{{{{{\rm{n}}}}}}/(1+{{{{{\rm{n}}}}}}/{{{{{\rm{N}}}}}})$ (refer to the main text and Fig. 2f). We used the parameter *N*, the ensemble size at which the IMSE reaches half of its asymptotic value, to characterize how quickly a given neuronal ensemble approaches saturation. The main text notes the ensemble size at which the slope on the fitted curve is 5% of ${{{{{{\rm{I}}}}}}}_{0}$. This ensemble size is equivalent to $\,(\sqrt{20}-1){{{{{\rm{N}}}}}}$ or $\sim\! 3.47{{{{{\rm{N}}}}}}$. To aggregate these values across sessions and animals, we first averaged sessions from the same animal and then reported the range across animals. We used a geometric mean to mitigate potential outliers. Using the geometric mean over each animal, the spatial accuracy reached 5% of its original slope at [346 - 1402] neurons. As another measure of saturation, we computed the ensemble size needed to reach 95% of the asymptotic IMSE, equivalent to 19 *N*. In that case, the values were [1894–7671] neurons.

### Dimensionality reduction of neural data

To visualize high-dimensional neural activity data on a low-dimensional plot, we used partial least squares (PLS) regression^70^. PLS found the dimensions in the space of ensemble responses most highly correlated with the spatial position and direction of motion variables. PLS regression is particularly suited when the estimation matrix has more neurons than observations (trials) and correlations between the estimated values. The neural responses and both behavioral variables were *z*-scored before running PLS. For the plot in Fig. 3a, we did not cross-validate the PLS dimensions to show the behavior for the entire session on one set of axes because we did not rely on PLS to provide an unbiased estimate of the spatial accuracy. Instead, we only used the PLS dimensions to visually contrast the noise covariance structures between the ensemble andshuffled ensemble data. We later cross-validated the PLS in several 2-D and 3-D subspaces to confirm that the description in Fig. 3a was accurate.

### Quantifying the signal and noise overlap

To determine how noise correlations influenced the extent to which trial-by-trial fluctuations overlapped with the position signal, we compared overlap between signal direction and each of the noise covariance matrix’s principal eigenvectors.

#### Definitions

“signal direction”*-* To approximate the direction tangent to the neuronal population tuning curve, ${{{{{\bf{f}}}}}}^{{{\prime} }}$, we defined the signal vector $\Delta \vec{{{{{{\rm{\mu }}}}}}}$ as the vector pointing from the trial-averaged population vector of one spatial bin to the one adjacent to it. The corresponding unit vector gives the signal direction. With 20 spatial bins, we have 19 such vectors per direction of motion.

“noise correlations”—We defined the neuronal activity fluctuations in response to a spatial position and direction of motion as the activity at each trial minus the trial-averaged activity at that position with the same direction of motion. For example, suppose the neuronal activity in neuron *n*, at spatial-directional bin *k*, and trial *t* is, in that case, the corresponding activity fluctuation is ${X}_{{nkt}}- < {X}_{{nkt}}{ > }_{t}$, where $< \,{\cdot > }_{t}$ denotes trial averaging.

“noise covariance matrix”- For each spatial bin, we counted each of the mean-subtracted population vectors from different trials as samples of the noise distribution. We then calculated the covariance matrix and its principal eigenvectors for unmodified and shuffled data. These principal eigenvectors are referred to as the noise directions.

“the overlap”*-* For each of the noise directions in a given spatial bin, we calculated the dot-product with the signal direction from the same bin to the next one (in each corresponding running direction), representing the angle’s cosine between the two unit vectors. Then, as an empirical estimate, in Fig. 4b, we used the difference between the sum of squares of the cosine angles up to the sixth noise direction in the real and shuffled data to quantify by how much the correlations increased the overlap of noise directions with signal direction.

### Analyzing signal and noise in the spatial code

To observe how signal and noise regarding spatial encoding change as a function of ensemble size, we first sought to project the neural activity onto the signal direction and then analyze the signal and noise within this one-dimensional subspace. We approximated the amount of signal in the space of population firing rates as the squared difference between trial-averaged responses of adjacent spatial bins (${\left|\Delta \vec{{{{{{\rm{\mu }}}}}}}\right|}^{2}$). This quantity grew linearly with ensemble size and was independent of trial shuffling (Fig. 3b, c). Similarly, we defined noise ${\sigma }^{2}$ as the trial-by-trial variance of the neuronal population responses along the signal direction between two adjacent spatial bins (Fig. 3b). The noise in the unmodified data kept increasing linearly with ensemble size, whereas in the shuffled data, it reached saturation well within the first ~100 neurons (Fig. 3d).

To produce estimates of the true signal and noise without overfitting our data, we first used 50% of the trials to calculate the difference between mean responses of adjacent spatial bins ($\Delta {{{{{{\rm{\mu }}}}}}}_{{{{{{\rm{train}}}}}}}$ vectors). We then projected each spatial bin’s neural activity from the other 50% of the trials onto the direction of the $\Delta {\mu }_{{train}}$ vector between it and the following spatial bin. From this one-dimensional projection, we found ${\left|\Delta {{{{{{\rm{\mu }}}}}}}_{{{{{{\rm{test}}}}}}}\right|}^{2}$ and ${{{{{{\rm{\sigma }}}}}}}_{{{{{{\rm{test}}}}}}}^{2}$ as the squared differences between the mean responses of the adjacent spatial bins and the pooled variances of both bins. Finally, we computed their median values over all adjacent spatial bin pairs (Fig. 3c, d).

In formulaic terms, we estimated signal and noise quantities as follows: Let ${{\hat{\Delta} {{{{{\rm{\mu }}}}}}}}_{{{{{{\rm{train}}}}}}}$ be the signal direction estimated from half of the trials, and let ${\Delta {{{{{\rm{\mu }}}}}}}_{{{{{{\rm{test}}}}}}}$ and ${\Sigma }_{{{{{{\rm{test}}}}}}}$ represent the difference in mean responses between adjacent spatial bins and the pooled covariance matrix from both bins, respectively, both calculated on the remaining half of the trials. From these variables, we estimated signal quantity (or ${{{{{{\rm{|}}}}}}\Delta {{{{{\rm{\mu }}}}}}{{{{{\rm{|}}}}}}}^{2}$) as $({\hat{\Delta}} {{{{{\rm{\mu }}}}}}_{{train}}^{T}.{{\Delta} {{{{{\rm{\mu }}}}}}}_{{test}})^{2}$, and noise quantity (or$\,{\sigma }^{2}$) as ${{\hat{\Delta} {{{{{\rm{\mu }}}}}}}}_{{train}}^{T}{\Sigma }_{{test}}{{\hat{\Delta} {{{{{\rm{\mu }}}}}}}}_{{test}}$.

We repeated the calculations for 80 random subsets of neurons at different ensemble sizes and calculated the values of ${{{{{{\rm{|}}}}}}\Delta {{{{{\rm{\mu }}}}}}{{{{{\rm{|}}}}}}}^{2}$ and ${\sigma }^{2}$ from the unmodified and shuffled data (Fig. 3c, d). We computed the linear slope values of ${{{{{{\rm{|}}}}}}\Delta {{{{{\rm{\mu }}}}}}{{{{{\rm{|}}}}}}}^{2}$ and ${\sigma }^{2}$ as functions of ensemble size using values from ensemble sizes of over 100 cells to ensure that only the linear regime of the curve participated in the calculation of the slopes. We repeated this analysis on the responses of each cell individually rather than a population projection. For each cell, from the median values of ${{{{{{\rm{|}}}}}}\Delta {{{{{\rm{\mu }}}}}}{{{{{\rm{|}}}}}}}^{2}$ and ${\sigma }^{2}$ over spatial bins, we defined the ratio ${{{{{{\rm{|}}}}}}\Delta {{{{{\rm{\mu }}}}}}{{{{{\rm{|}}}}}}}^{2}/{\sigma }^{2}$ as the signal-to-noise ratio (SNR) of the cell. We found that the average SNR across all cells correlated with the initial linear slope of decoding accuracy (${{{{{{\rm{I}}}}}}}_{0}$) when comparing across mice (Suppl. Fig. 11b). We repeated the above analysis to calculate the SNR along each noise component (Fig. 4a). Still, instead of estimating the signal direction from the training set (as ${{\hat{\Delta} {{{{{\rm{\mu }}}}}}}}_{{train}}^{T}$), we estimated the principal components of the noise covariance matrix from the training set (${{{{{\rm{P}}}}}}{{{{{{\rm{C}}}}}}}_{k,{train}}$) and calculated the SNR as described above after projecting the testing set onto each ${{{{{\rm{P}}}}}}{{{{{{\rm{C}}}}}}}_{k,{train}}$ direction. As a reference, we also included the SNR from projecting the training set itself onto each ${{{{{\rm{P}}}}}}{{{{{{\rm{C}}}}}}}_{k,{train}}$ direction to show the effect of cross-validation on the estimates of SNR along the noise principal components. Note that the sum of SNR values along each PC is equal to the SNR along the direction maximizing SNR, which is generally not identical to the SNR along the signal direction.

### Signal and noise analysis for the direction of motion variable

The definitions of signal (${{{{{{\rm{|}}}}}}\Delta {{{{{\rm{\mu }}}}}}{{{{{\rm{|}}}}}}}^{2}$) and noise (${\sigma }^{2}$) for the direction of motion variables are identical to those of the position variable, except that, instead of being measured between two adjacent spatial bins, they are measured between bins with the same spatial position but opposite directions of motion.

First, we held out 50% of the trials as a test set, with the remaining being the training set. Next, we determined the direction of motion's signal direction for a given spatial position as the mean population vector of the rightward bin minus the mean of the leftward bin at that position, all using the training set data. We then projected the test data onto the signal direction and calculated ${{{{{{\rm{|}}}}}}\Delta {{{{{\rm{\mu }}}}}}{{{{{\rm{|}}}}}}}^{2}$ and ${\sigma }^{2}$ using the right and left bins for each discrete spatial position value. ${{{{{{\rm{|}}}}}}\Delta {{{{{\rm{\mu }}}}}}{{{{{\rm{|}}}}}}}^{2}$ is the squared distance between the means of the left and right bins, and ${\sigma }^{2}$ is the pooled variance from both bins.

### Path analysis

We employed path analysis to show that task performance best correlated with asymptotic decoding performance, unmediated by the number of trials performed (Suppl. Fig. 8d)^71^. The analysis included number of trials and task performance as predictors in a linear model of the log asymptotic IMSE. The path analysis used one sample per session from the sessions with over 200 recorded neurons (*n* = 73 sessions).

### A synthetic PF model

To illustrate the PF shape variability, we used a synthetic representation of spatially-tuned neurons covering the spatial environment in our task. To generate artificial populations of cells tesselating the entire linear track, we created Gaussian-like receptive fields located randomly within the track represented by 20 individual segments. For the “narrow” distribution, we generated gaussian PFs with a log-normal distribution of half-widths with mean equal to 0 and variance equal to 0.3 [log(bins)]. For the broad distribution of half-widths, the mean was equal to 0, and variance equal to 2 [log(bins)]^38^. The variance of the slope distribution for 50 synthetic neurons across all bins was then computed. This procedure was repeated 1000 times to obtain the distribution of the *normalized signal variance* (see below) for these two types of PF distributions (“narrow” and “broad”) (Fig.4c, d).

### Definition of the normalized signal variance (NSV)

To quantify the degree to which the spatial (or directional) signal direction was distributed across the neuronal ensemble, we took the signal direction vector ${\hat{\Delta} {{{{{\rm{\mu }}}}}}}=\vec{\Delta {{{{{\rm{\mu }}}}}}}/{{{{{\rm{|}}}}}}\vec{\Delta {{{{{\rm{\mu }}}}}}}{{{{{\rm{|}}}}}}$ and found the variance of its squared elements, termed the normalized signal variance (NSV).

$${{{{{\rm{NSV}}}}}}={{{{{\rm{VA}}}}}}{{{{{{\rm{R}}}}}}}_{{{{{{\rm{i}}}}}}}[({{\hat{\Delta}} {{{{{\rm{\mu }}}}}}})_{{{{{{\rm{i}}}}}}}^{2}]$$

We used signal direction only and not the original ${\vec{{\Delta}} {{{{{\rm{\mu}}}}}}}$ because the overall magnitude varies enormously across spatial bins and does not affect the degree of distributivity of the code.

### Statistics

We have also utilized common tests not described in the Method sections. The Wilcoxon signed-rank test was  used as a paired difference test without the assumption of normally distributed samples. We utilized the Pearson correlation coefficient $\rho$ and p-values to describe linear correlation between two data sets, and the coefficient of determination r^2^ to describe the power of the model.

### Reporting summary

Further information on research design is available in the Nature Research Reporting Summary linked to this article.

## Supplementary information

Supplementary Information

Reporting Summary

## Data Availability

Data from all figures are provided in the supplementary material file called: “SourceData” file. All cells raw data are available at CRCNS—Collaborative Research in Computational Neuroscience (10.6080/K0GH9G5X).

## Code availability

Neuronal activity data analysis was performed using our own codes in Matlab. The codes are available at: https://github.com/approbatory/ML-project.
