# Supplementary material for: Noise correlations in neural ensemble activity limit the accuracy of hippocampal spatial representations
Source: Nat Commun. 2022 Jul 25;13:4276. doi: 10.1038/s41467-022-31254-y (PMC9314334; doi:10.1038/s41467-022-31254-y)
Supplement: Supplementary file 1 — Supplementary Information [file 41467_2022_31254_MOESM1_ESM.pdf]

Supplementary material

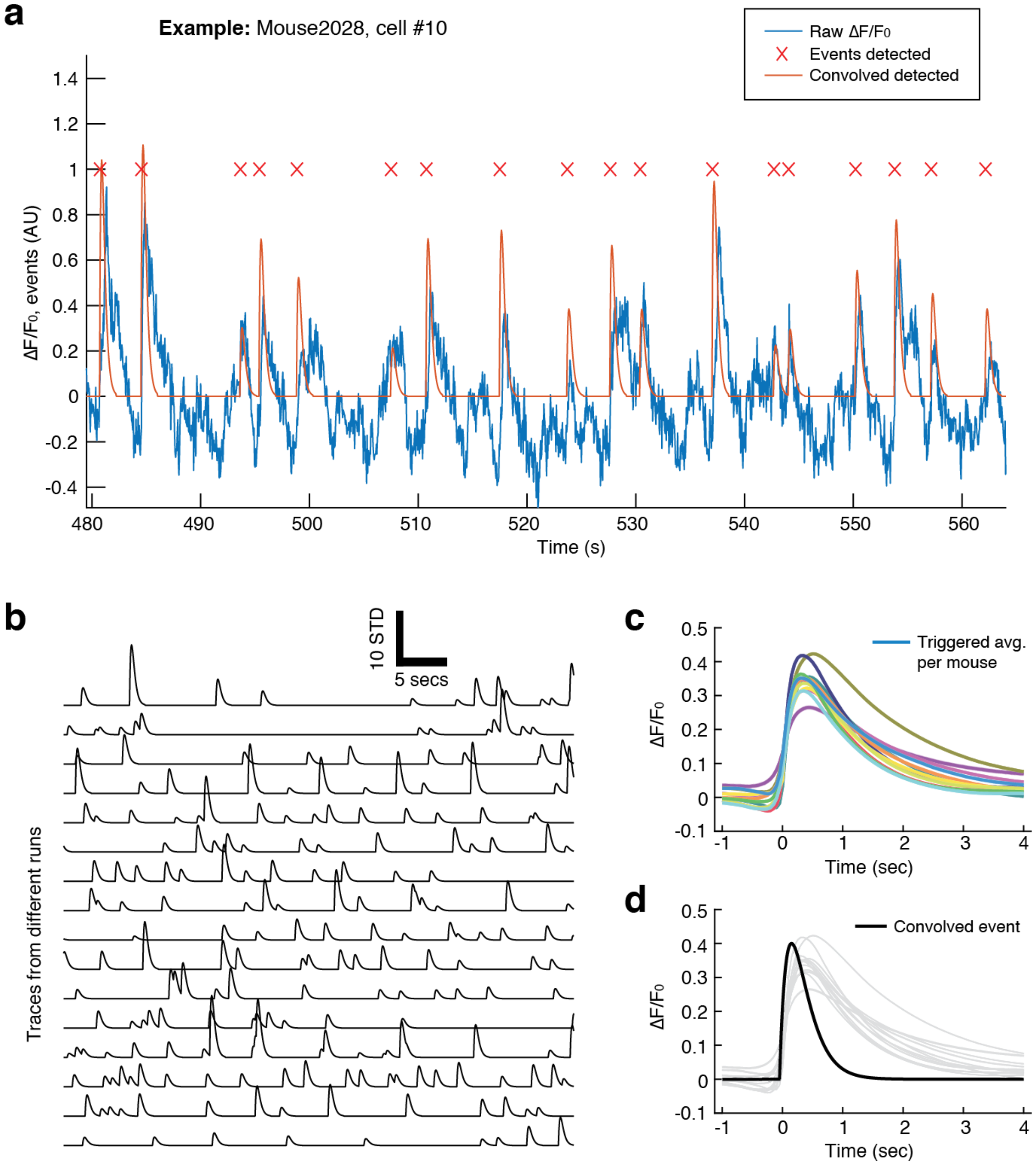

Supplementary Figure 1: Examples of event detection and comparison with calcium traces. a) The algorithm, explained in the methods section, provides time frames of events based on the dynamics of the rising phase of the calcium activity recorded during freely moving behavior. Convolution with an alpha function is needed to obtain a continuous trace to implement position decoding, analyze noise principal components, and derive the geometry between signal and noise components. **b**) Responses of 16 different neurons over the same time period after applying the event detection algorithm. The value of the event trace corresponds to the summation over time of different convolved events. **c**) Showing the detected event-triggered average over the calcium traces to identify the actual rise and decay times. Each color represents one of the 12 animals. Thus, the rise and decay times are similar among recordings. **d**) Events are convolved with an alpha function $\alpha(t) = (t/\tau)\exp(1 - t/\tau)$ with the time constant τ =0.2sec. Our deconvolved events have a similar rise time to the calcium traces, corresponding to the kinetics of the indicator (Chen *et al.* 2013), but a 3-4 times shorter decay time to avoid spillover of signal affecting neighboring spatial bins during runs in the linear track (black vs. grey traces).

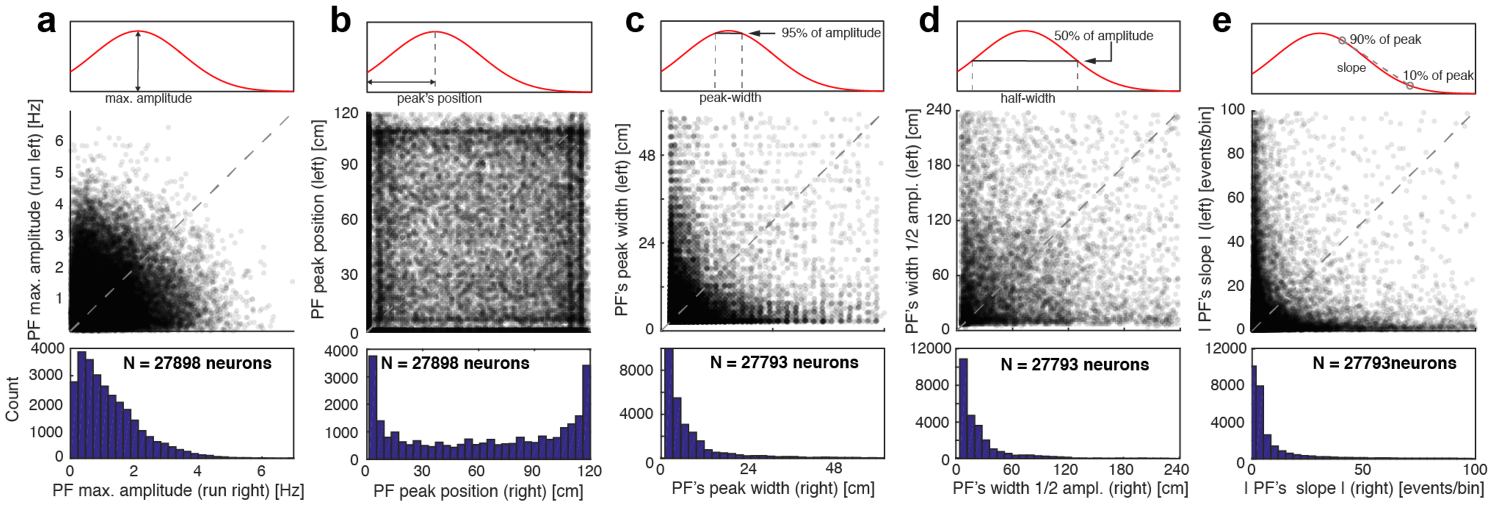

Supplementary Figure 2: Characterization of the place fields in our data set. a) Distribution of the PFs' maximum amplitude in number of events/sec. **b**) Distribution of the PFs' peak along the track, shown jointly for the leftward and rightward motion PFs, in all recorded cells (N=27898). The off-diagonal high density evinces a remapping of the same cells depending on the directionality. **c**) The PFs' peak width for all place cells recorded (N=27793) with a minimal spatial modulation (**Definition:** Max firing rate > 20% of the Sta. Dev. of the response on the track). **d**) The PFs' half-width for all place cells recorded. **e**) The distribution of slopes for all place cells recorded. Using a *slope-code,* the brain profits from the spatial stimulus's continuity, detecting response changes in a given position's vicinity. However, errors when estimating the slope would also reduce accuracy.

Supplementary Table 1: Comparison of place field characteristics among previous studies.

Author	Species, Recording Method, Behavior	mean firing rate (Hz)	PFs' inside/outside firing rate (Hz)	Maximum rate (Hz)	PF width ½-ampl. (cm)	Trial-by-trial variability *	Spatial Information bits/spikes **	Single-cell signal-to-noise ratio ***
**Frank et al. 2000**	Rat, Ephys, W-Maze	1.1± 1.1		21.9 ± 12.5	~25		2.34 ± 1.25	
**Lee et al. 2004**	Rat, Ephys, Circular maze	1.1 ± 0.1	3.9 / ~	8.7 ± 0.5				
**Kelemen and Fenton 2010**	Rat, Ephys, Circular aversive rotation arena	[0-2]						
**Cabral et al. 2014**	Mouse, Ephys, circular track				85		0.51± 0.05 (sess. 1,2) 0.54± 0.06 (sess. 9,10)	
**Stefanini et al. 2020**	Mouse, calcium-imaging, open field	0.06 ± 0.04						
**Raveau et al. 2018**	Mouse, Ephys, linear-track (120cm long)	0.45 ± 0.43	0.68 ± 0.63 / 0.13 ± 0.05	1,2 ± 1	51.6 ± 49	0.39 ± 0.37	0.23 ± 0.42	1.4 ± 6.7
**Mizuseki et al. 2014**	Rat, Ephys, linear-track (250 cm long)	3.4 ± 5.8	5.7 ± 6.9 / 1.2 ± 0.36	9.3 ± 11	72 ± 73	4.9 ± 4.5	0.19 ± 0.054	0.43 ± 0.26
**Our Data**	Mouse, calcium-imaging, linear-track (120cm long)	0.33 ± 0.24	0.88 ± 0.72 / 0.034 ± 0.07	1.4 ± 0.72	37.8 ± 48.5	1.2 ± 0.66	0.24 ± 0.051	0.41 ± 0.34

* trial-by-trial variability: is the variance of the spike count over different visits of the animal to each per spatial bin, averaged over all spatial bins.

** Spatial information: is the $\frac{1}{N}\sum_{i=1}^{N} p_i . log_2(p_i)$, where p_i is the probability of firing a spike in bin i .

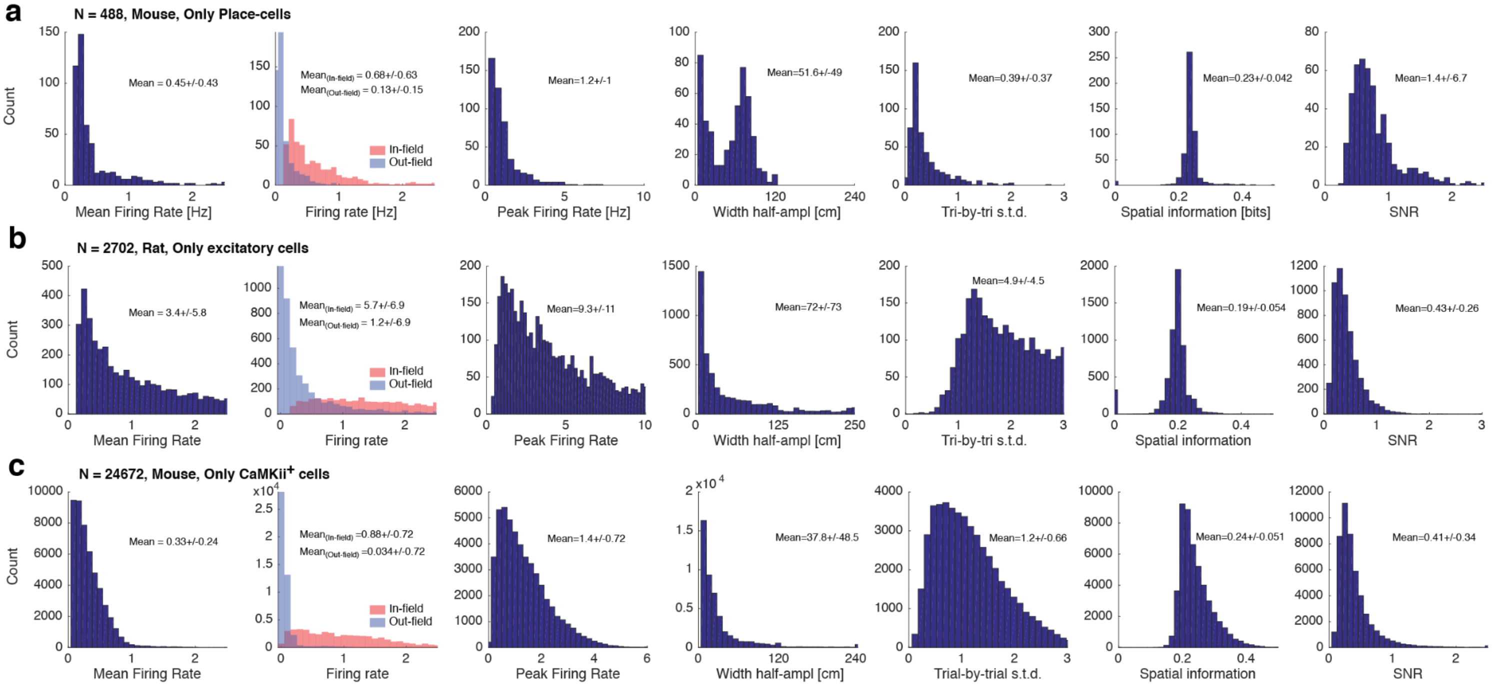

*** Single-cell signal-to-noise ratio: is the variance of the place field over the entire track (signal), divided by the mean trial-by-trial variability over the entire track (noise) as in (Stringer *et al.*, 2021).

Supplementary Figure 3: Detailed comparison between place field characteristics of three different data sets. Our analysis of the three data sets, including our own, shows consistency among different experimental recording techniques and animal species. Showing data sets from Mizuseki *et al.* 2014 (a), Raveau *et al.* 2018 (b), and our data (c). Despite the interspecies and inter-methodology differences, the spatial-information and signal-to-noise ratio (SNR), which accounts for the information carried by each neuron, is similar among all three data sets. The leftmost column was produced using all cells. The rest of the columns were generated using cells whose total spike count was higher than 100 events/spikes for every 10 min of recording. All place-fields characterization parameters were obtained after fitting a gaussian curve to the firing rates from different spatial bins. Notice that all three analyses were conducted on slightly different cell populations: Only place cells, Only excitatory cells (based on firing rate and spike shape), and only CaMKii cells transfected by the virus, in a), b) and c) respectively.

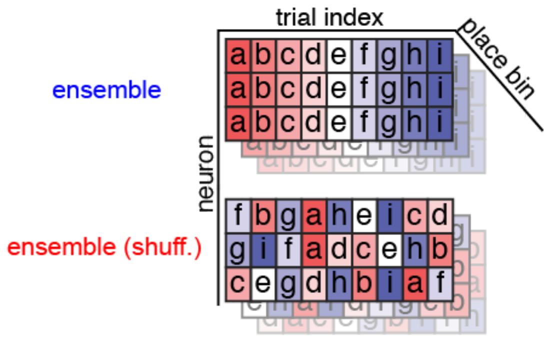

Supplementary Figure 4: Noise correlation elimination method. Schematic of the neural responses organized as a tensor of a neuron's mean activity at a particular spatial bin during an individual trial. The decoder predicts the mouse's spatial location and direction of motion based on the neural activity tabulated in each column of the tensor. To determine the effect of noise correlations on spatial representation, we created trial-shuffled versions of the neural recordings, we randomly permuted each cell's activity levels across trials (Bottom), breaking the simultaneity between them.

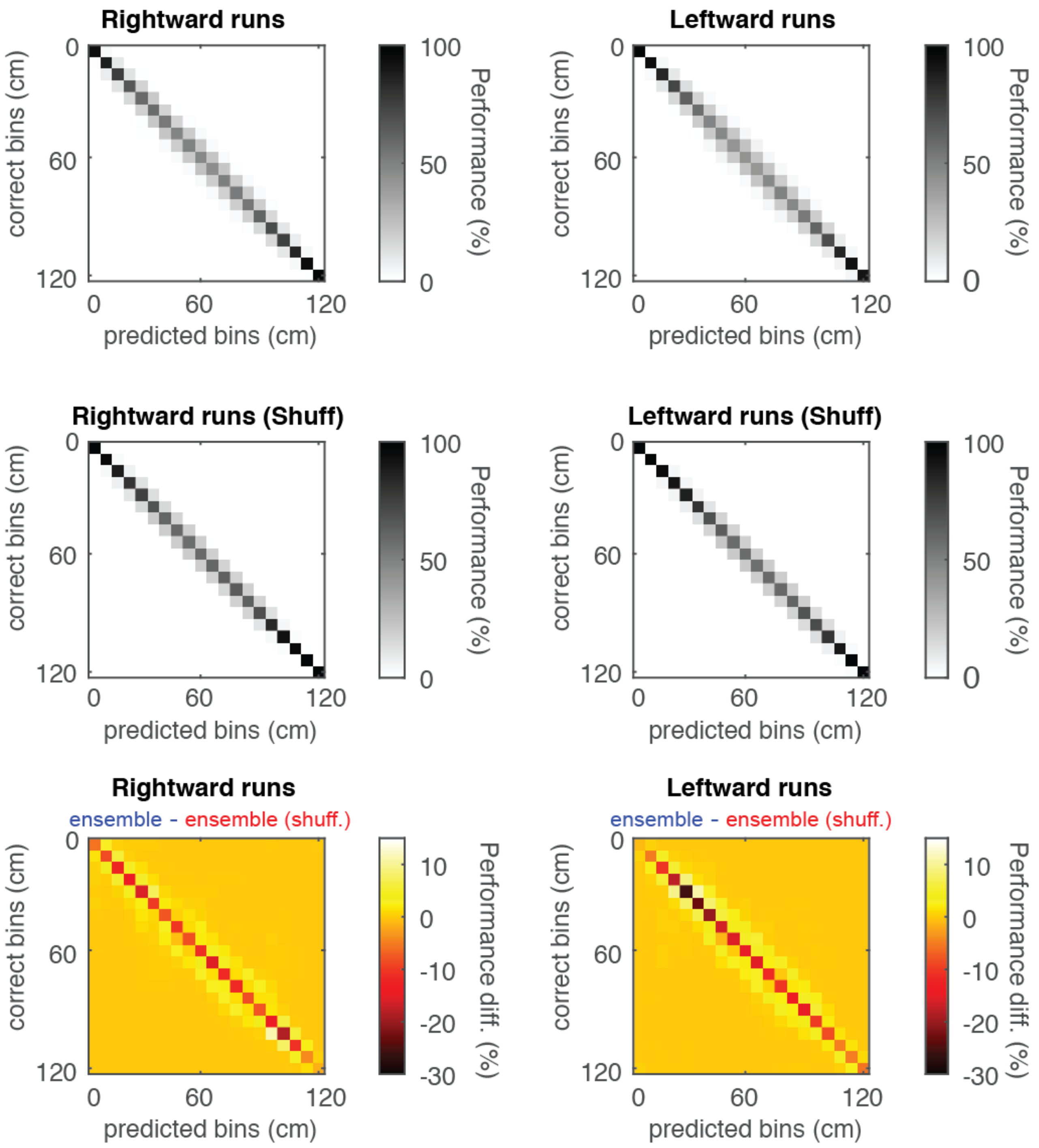

Supplementary Figure 5: Decoding errors in the unmodified and noise-correlation-free data. The confusion matrix for the ensemble and trial-shuffled data, using the same session as Figure 2c. Each matrix corresponds to the confusion matrix for rightward runs (left column) or leftward runs (right columns). The first row represents the confusion matrices for unmodified ensemble data. The middle row represents the matrices for the noise-correlations-free data. Since the errors were small in the first two sets of matrices, we computed the differences (unmodified - shuffled, final row) to observe the effect of noise correlations in different bin locations. Notably, all correct classifications (diagonals) were more common in the shuffled data, and nearly all misclassifications (off-diagonals) were more common in the unmodified data or otherwise equal.

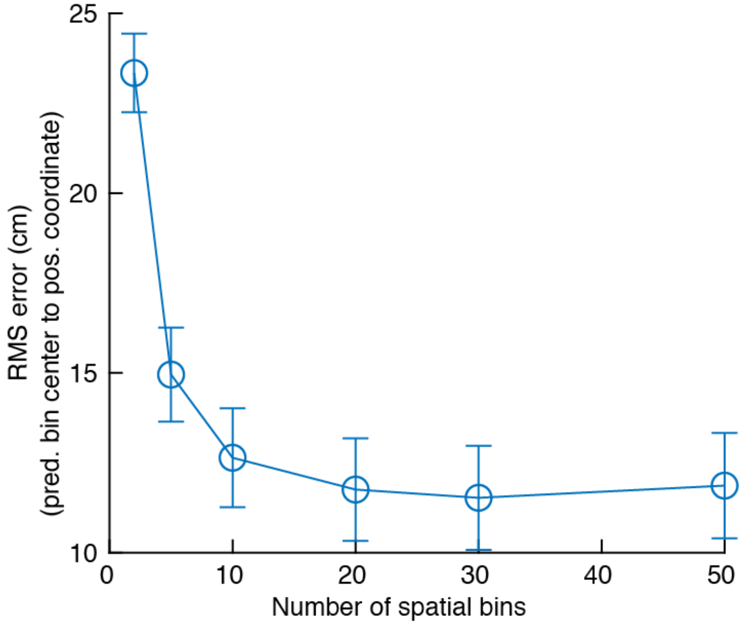

Supplementary Figure 6: Study of optimal bin size to obtain the smallest decoding error. We show the decoding error using different spatial bins to discretize the linear track. We ran the decoders using 80 train/test divisions per session, using 110 sessions and 12 mice. The error bars are the 95% confidence intervals. We utilized 20 bins throughout the article because it provided close to the smallest mean RMS error with a higher sample number per bin, which allowed us to study the geometric relation between noise structure and signal with higher statistical power. For 20 bins, the RMS error across sessions was 11.75 cm with a 95% CI of [10.33 - 13.18 cm] based on the SEM. For 30 bins, the RMS error was 11.53 cm with a 95% CI of [10.08 - 12.98 cm]. To make the comparison between discretization schemes fair, we computed the RMS error relative to the continuous, non-discretized position variable, unlike in Figure 2.

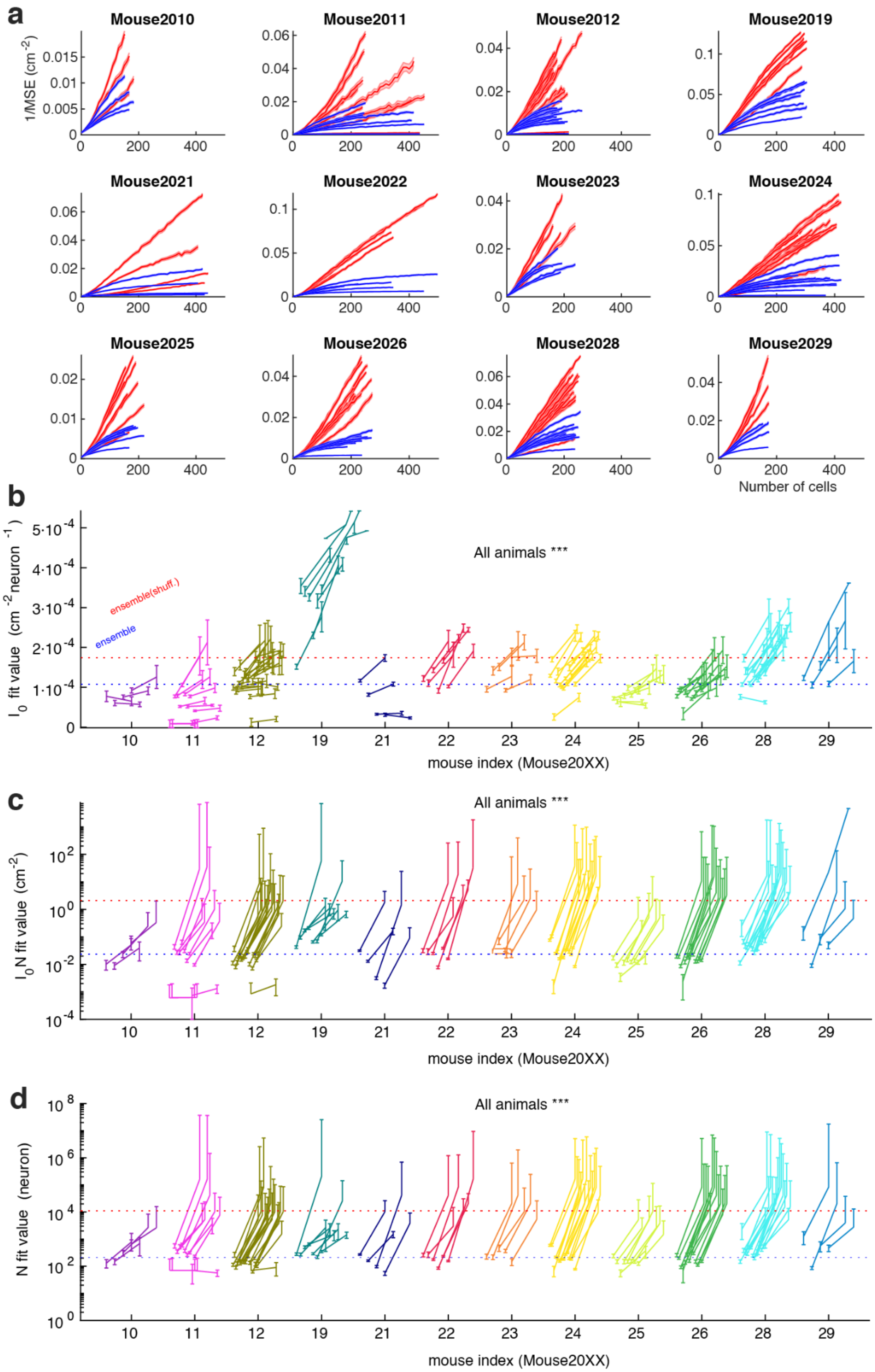
a
Mouse2010
Mouse2011
Mouse2012
Mouse2019
Mouse2021
Mouse2022
Mouse2023
Mouse2024
Mouse2025
Mouse2026
Mouse2028
Mouse2029
1/MSE (cm -2)
Number of cells
b
I0 fit value (cm -2 neuron -1)
ensemble(shuff.)
ensemble
All animals ***
mouse index (Mouse20XX)
c
I0N fit value (cm-2)
All animals ***
mouse index (Mouse20XX)
d
N fit value (neuron)
All animals ***
mouse index (Mouse20XX)

Supplementary Figure 7: Noise correlations limit the spatial information encoded in the neural ensemble. Display of the data for different animals and sessions. (**a**) Inverse mean squared error (IMSE) as a function of the number of cells included in the decoder for all sessions from each animal. The red curves are above the blue curves for each corresponding session pair. The shaded regions represent 95% confidence intervals over random subsets of neurons trained with half of the trials and tested with the other half. (**b**) The value of I_0 for all sessions from each animal. The color code for mouse identity is as in Suppl. Fig. 11a. The plot shows the initial linear slope values for unmodified (left) and shuffled (right) data. There was a significant difference across all sessions due to a sublinear increase in accuracy for small ensemble sizes (Wilcoxon signed-rank test, $P < 0.001$). The sublinear growth is an artifact of a low sampling of the spatial bins due to the low number of neurons for small ensemble sizes. Without this artifact, the two data sets should display equivalent I_0. Error bars are the 95% confidence intervals for the fitting of IMSE over random subsets of neurons trained with half of the trials and tested with the other half. (**c**) The fitted parameters I_0 and N product represent the projected asymptotic IMSE shown for the unmodified (left) and shuffled (right) data. In all but a few sessions, the shuffled data had a higher asymptotic IMSE. The values increased by one order of magnitude in the shuffled data (Wilcoxon signed-rank test, $P < 0.001$). Across animals, task performance explained the differences in asymptotic IMSE (Suppl. Fig. 8). Error bars as in **c**. (**d**) The value of N for the two data sets. N is significantly larger for the data with no correlated fluctuations (Wilcoxon signed-rank test, $P < 0.001$) Error bars as in **d**.

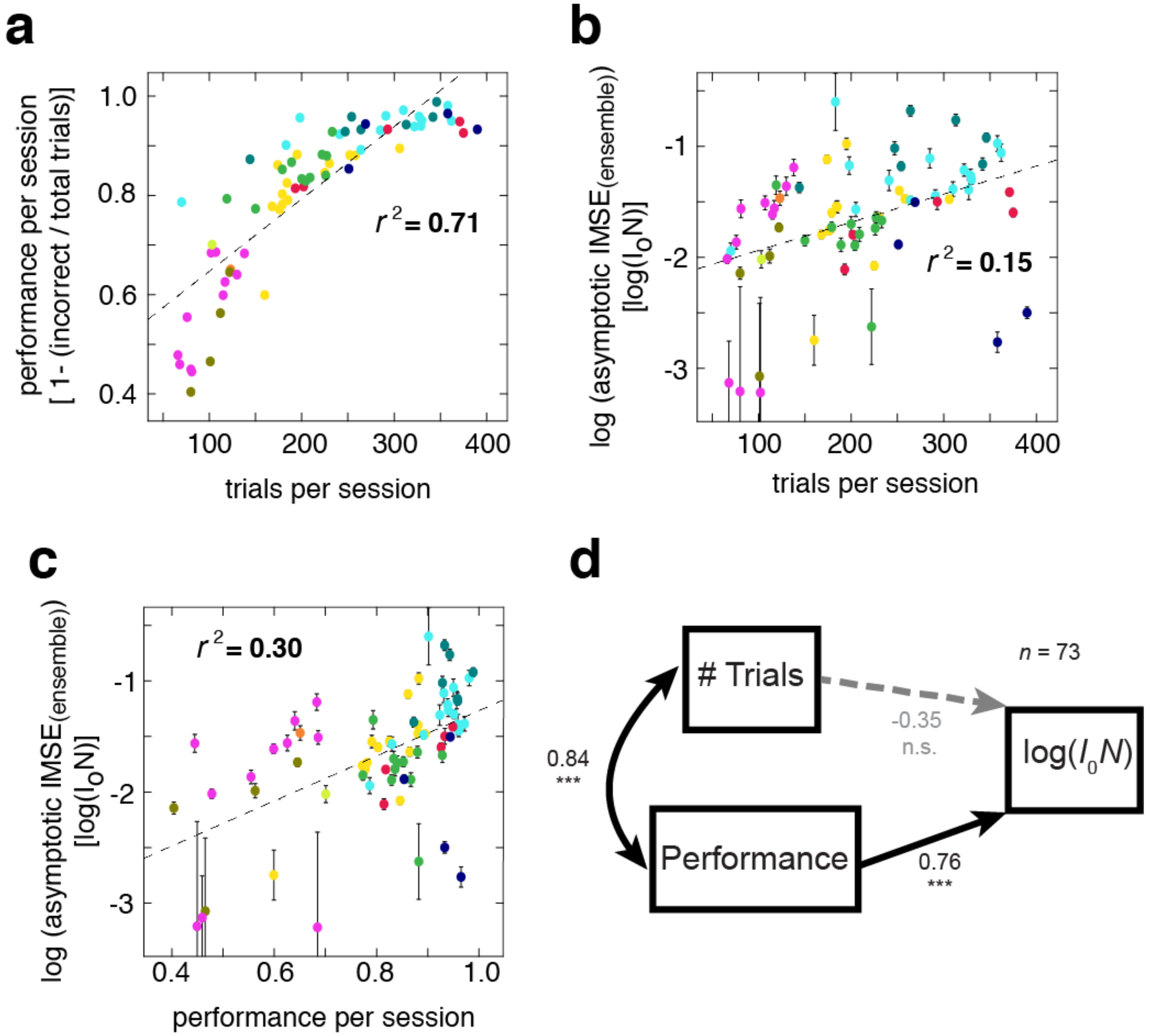

Supplementary Figure 8: Task performance correlated with decoding accuracy. a) Performance, computed as the ratio of correct over total trials, correlated with the number of trials done by an animal on a given session, using n = 73 sessions (r^2 = 0.71, Pearson-corr = 0.85, $p_{val} = 4x10^{-21}$). The color code for mouse identity is as in Suppl. Fig. 11a. **b)** The asymptotic decoding accuracy for large ensembles correlated with the number of trials done during the session (r^2 = 0.11, Pearson-corr = 0.34, p_{val} = 0.0029). Each dot corresponds to the average over different subsets of neurons, and error bars correspond to the 95% confidence interval. Trial number has a single value per session. **c)** The asymptotic decoding accuracy for large ensembles correlated with the performance per session (r^2 = 0.26, Pearson correlation = 0.52, $p_{val} = 2.1x10^{-6}$). Each dot corresponds to the average over different subsets of neurons, and error bars correspond to the 95% confidence interval. Trial number has a single value per session. **d)** Path analysis indicates that the performance significantly predicts the decoding accuracy when jointly considered, yet the number of trials is inversely correlated (not significantly). Therefore, the effect of differences in data size on our results was minimal compared to behavioral factors such as task performance. This analysis compared the variance between individual sessions (n = 73).

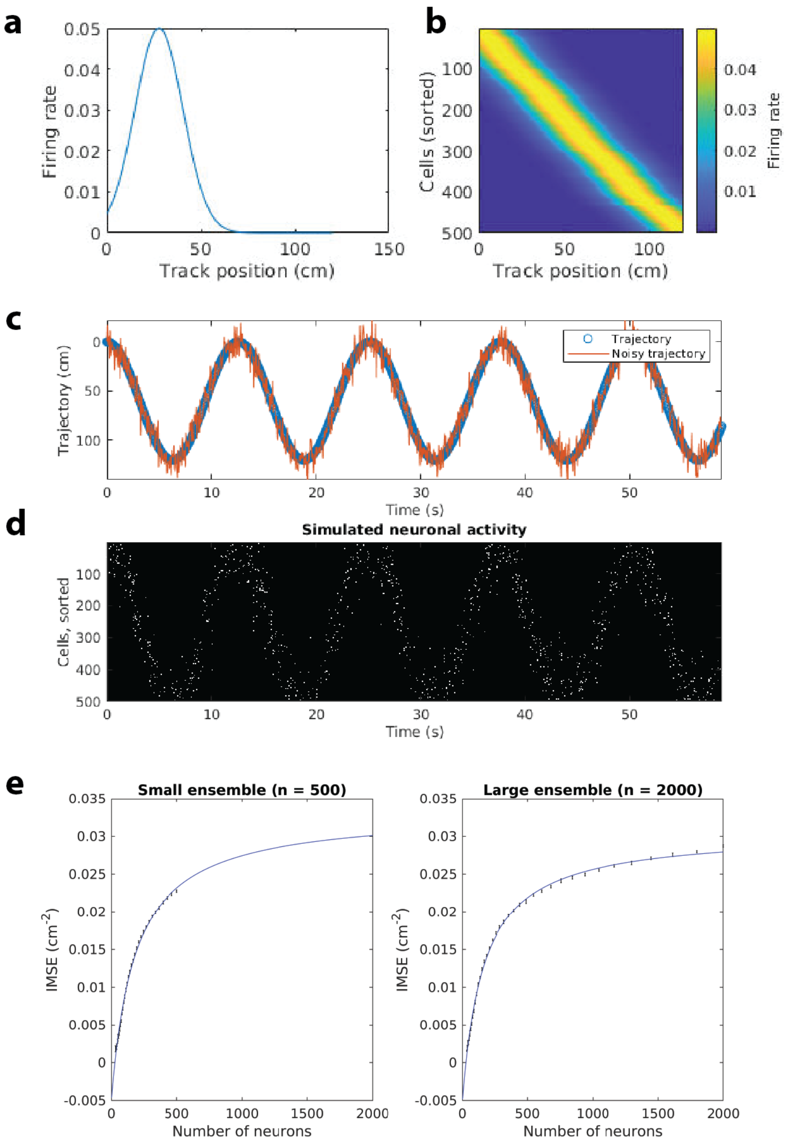

Supplementary Figure 9: Validation of the asymptote fitting method. a) A simulated place field for an example neuron. The neuron's firing rate is a gaussian function of the track position. The center of the place field for each simulated neuron is a random position on the track, drawn uniformly. The full-width at half maximum (FWHM) is set to 30 cm for all the neurons. The maximal firing rate of each neuron is set to 0.05. **b)** A heatmap of the firing rate functions of all the neurons in the simulated ensemble, sorted by their preferred firing position as functions of the track position, sorted by their preferred firing position. With 500 neurons, the place fields tile the entire track. **c)** A small segment of a 30-minute simulated mouse trajectory on a linear track. They show the true trajectory (in blue) and the same trajectory corrupted by gaussian input noise with an Std. Dev. of 10cm (in red). Instead of using the position values of the true trajectory as the input to our simulated ensemble of independent Poisson neurons, we used the noise-corrupted trajectory to cause information-limiting noise correlations when trying to decode the true trajectory from the set of neuronal responses. Note that these do not represent decoded trajectories but rather the inputs into the generative model of neuronal activity. **d)** A sample of a set of simulated neuronal responses, sorted by the preferred firing position of each neuron. Each neuron fires according to an inhomogeneous Poisson random variable, dependent on the position, with a firing rate that follows the place field of that neuron (b). **e) Left:** decoding performance at various sub-ensemble sizes from a 500 neuron (black) ensemble. The decoding scheme, including cross-validation, follows the methods used in the paper, except for using a Poisson naive Bayes model in place of the SVM ensemble. The centers and error bars show the mean and the 95% CIs over 16 random subsets and train-test divisions, respectivaly. It also shows the parametric fit curve, extended up to 2000 neurons (blue). **Right:** The same results as in the left plot, but using an ensemble with 2000 neurons. The two ensemble sizes produce consistent results.

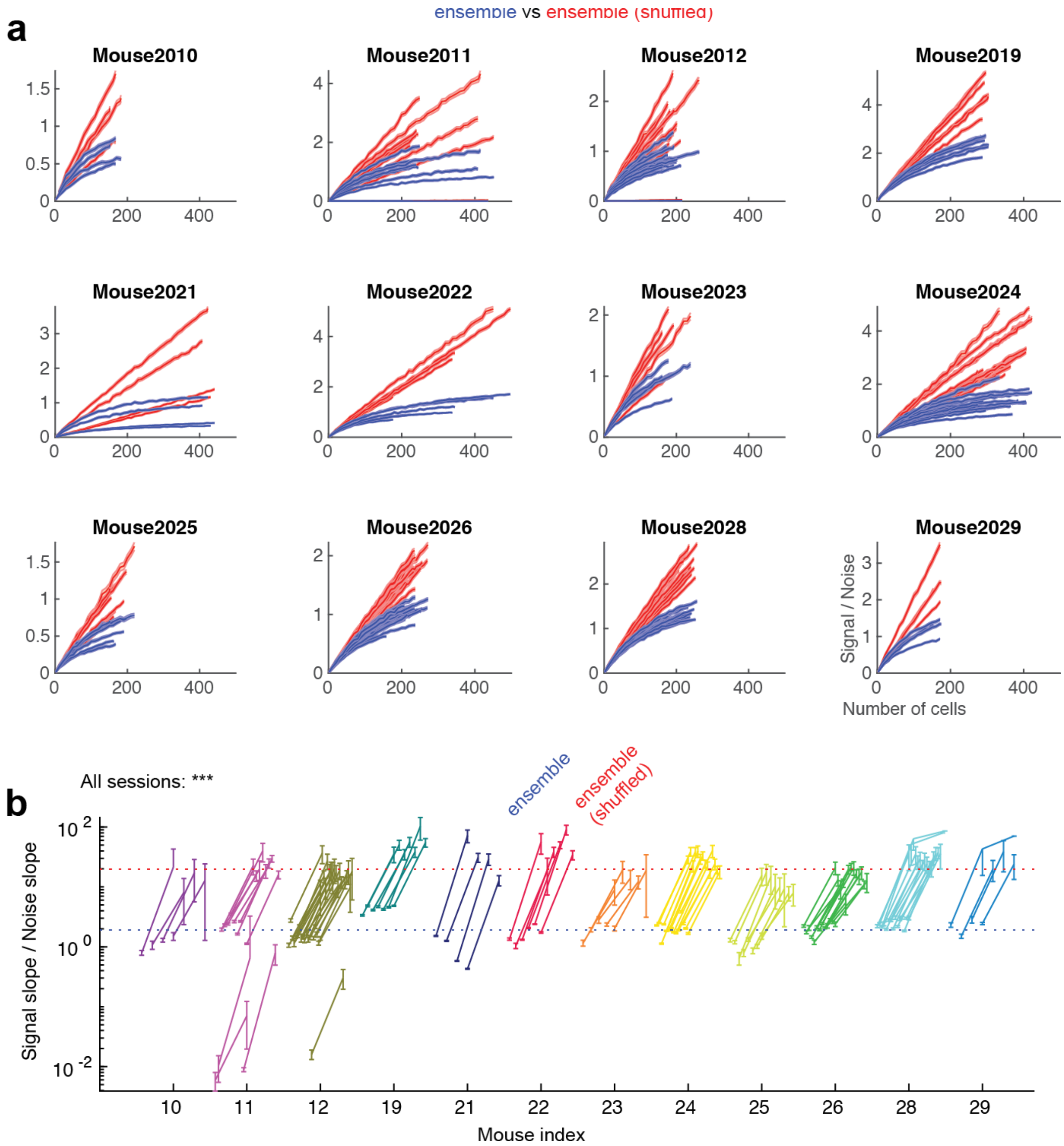

Supplementary Figure 10: The inferred asymptotic spatial SNR values were higher for the data without noise correlations. a) The signal-to-noise ratios (SNRs) as a function of ensemble size from the unmodified (blue) and shuffled (red) data, as in Figure 3**c**, are shown for all sessions and arranged by subject. Each value in the solid lines is the average over subsets of ensembles of different sizes. Shaded areas are the 95% confidence intervals. **b)** The slope of the signal curve divided by the slope of the noise curve as functions of ensemble size, comparing the unmodified (left endpoints) and shuffled (right endpoints) data, computed for ensemble sizes beyond 100 cells. Here we show each session individually and arranged by subject. The center of error bars are the average over the slopes fitted from different subsets of neurons from ensembles and the ensembles suffled. Error bars denote 95% confidence intervals. Dotted lines represent the median values over the real (blue) and shuffled (red) data. Shuffling significantly increased the values (Wilcoxon signed-rank test, $P < 0.001$). The color code for mouse identity is as in Suppl. Fig. 11a.

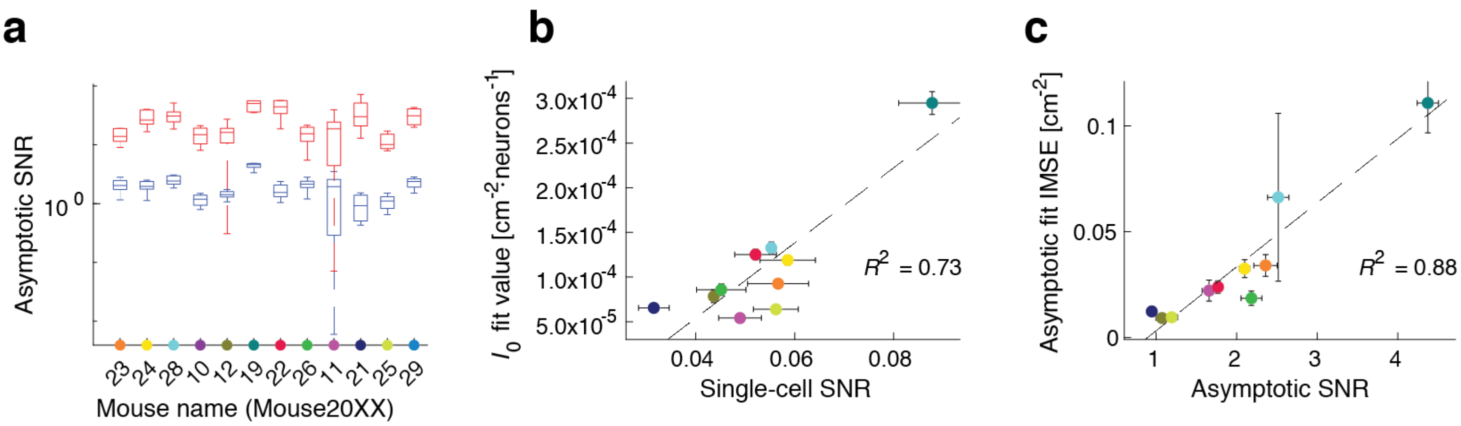

Supplementary Figure 11: Comparison of asymptotic SNR behavior across mice. (**a**) The estimated slopes of the "signal" curves from Figure 3d divided by the estimated slopes of the "noise" curves (excluding the first 100 neurons) are approximations of the asymptotic values of the SNR curves in Fig. 3e. They show asymptotic SNR values for the original (blue) and the shuffled (red) data. The values come from 110 sessions from 12 mice. The color code for animal names follows the labels on the *x*-axis. The code is the same for all three panels. The representation is on a logarithmic scale. On each box, the central mark is the median, the edges of the box are the 25th and 75th percentiles, and the whiskers extend to the most extreme data points not considered to be outliers (within 1.5 times the IQR from the 1st or 3rd quartiles). (**b**) The linear slope coefficients, I_0, from the IMSE of decoding, were strongly correlated with the SNR computed across each neuron individually, shown from 73 sessions from 10 mice with more than 200 neurons recorded per session, aggregated within each mouse. Each colored point corresponds to the average over sessions from each animal (color coded as in **a**). The error bars aggregate 95% confidence intervals from the I_0 fits. The variables were directly correlated across mice (r^2=0.73, p < 0.01). (**c**) The fitted asymptotic IMSE of the decoders (as in Fig. 2f), I_0N, correlated with the asymptotic SNR values. The plot shows data from the same sessions as (b), with the same color code and error bar definition. The variables were directly correlated across mice (r^2=0.88, $p < 4 \cdot 10^{-5}$). Each colored point corresponds to the average over sessions from each animal (color coded as in **a**). The error bars aggregate 95% confidence intervals from the fitted asymptotic IMSE.

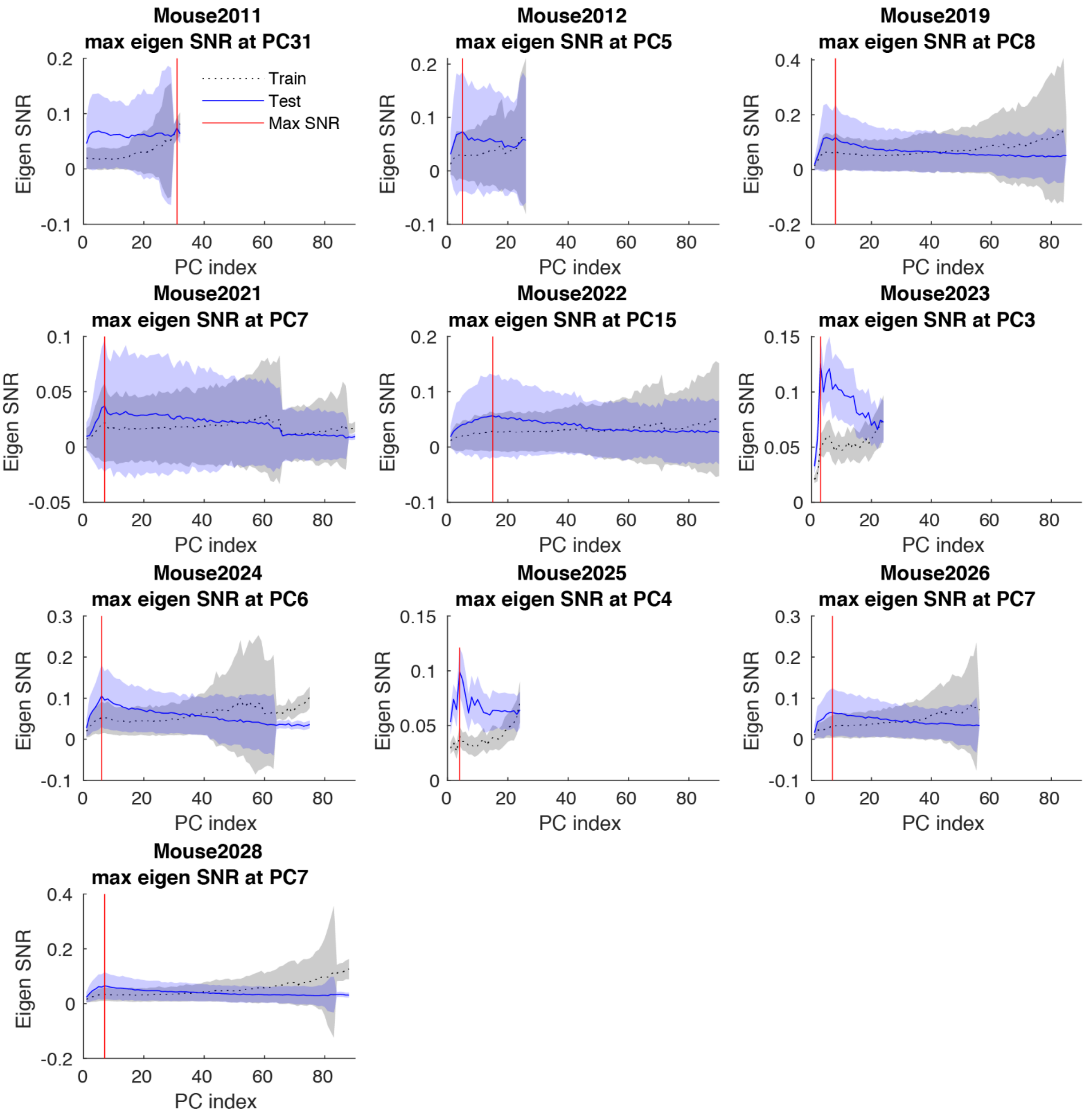

Supplementary Figure 12: The SNR along each noise principal component direction. The SNR along each noise covariance matrix eigenvector is shown separately for each mouse. Showing the SNR on the training set (dotted gray) and the testing set (solid blue). We plotted the median SNR over spatial bins for each mouse, averaged over 100 train/test sets, and aggregated over the available sessions ($n = 73$ sessions from 10 mice). We denote the dimensions with the highest SNR (red line). The shaded regions represent 95% confidence intervals based on the combined SEM across train-test splits and sessions.

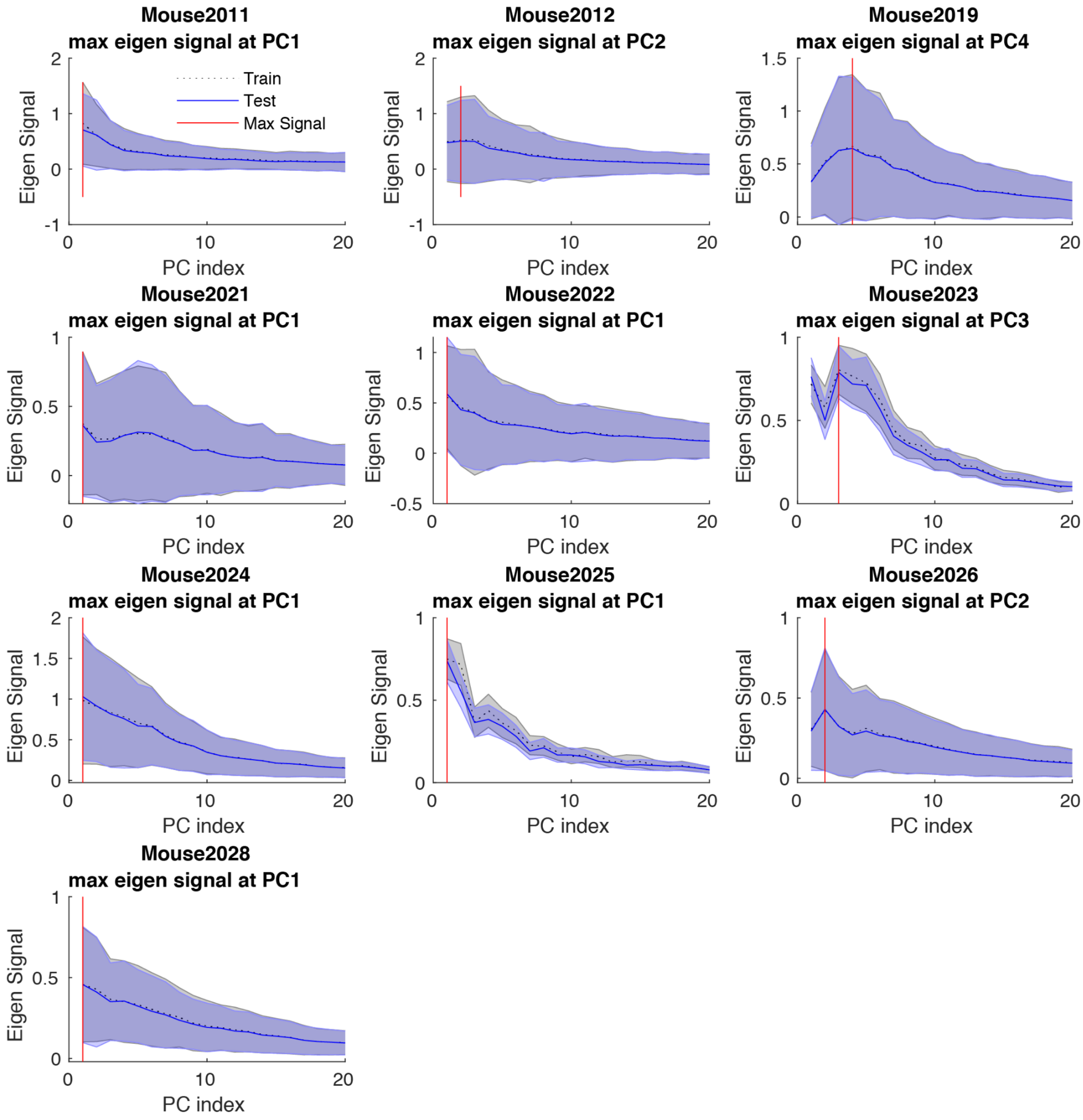

Supplementary Figure 13: The signal along each noise principal component direction. The signal along each noise covariance matrix eigenvector is shown separately for each mouse. Showing the signal on the training set (dotted gray, occluded) and on the testing set (solid blue). We plotted the median signal over spatial bins for each mouse, averaged over 100 train/test sets, and aggregated over the available sessions (n = 73 sessions from 10 mice). We denote the dimensions with the highest signal (red line). The shaded regions represent 95% confidence intervals based on the combined SEM across train-test splits and sessions.

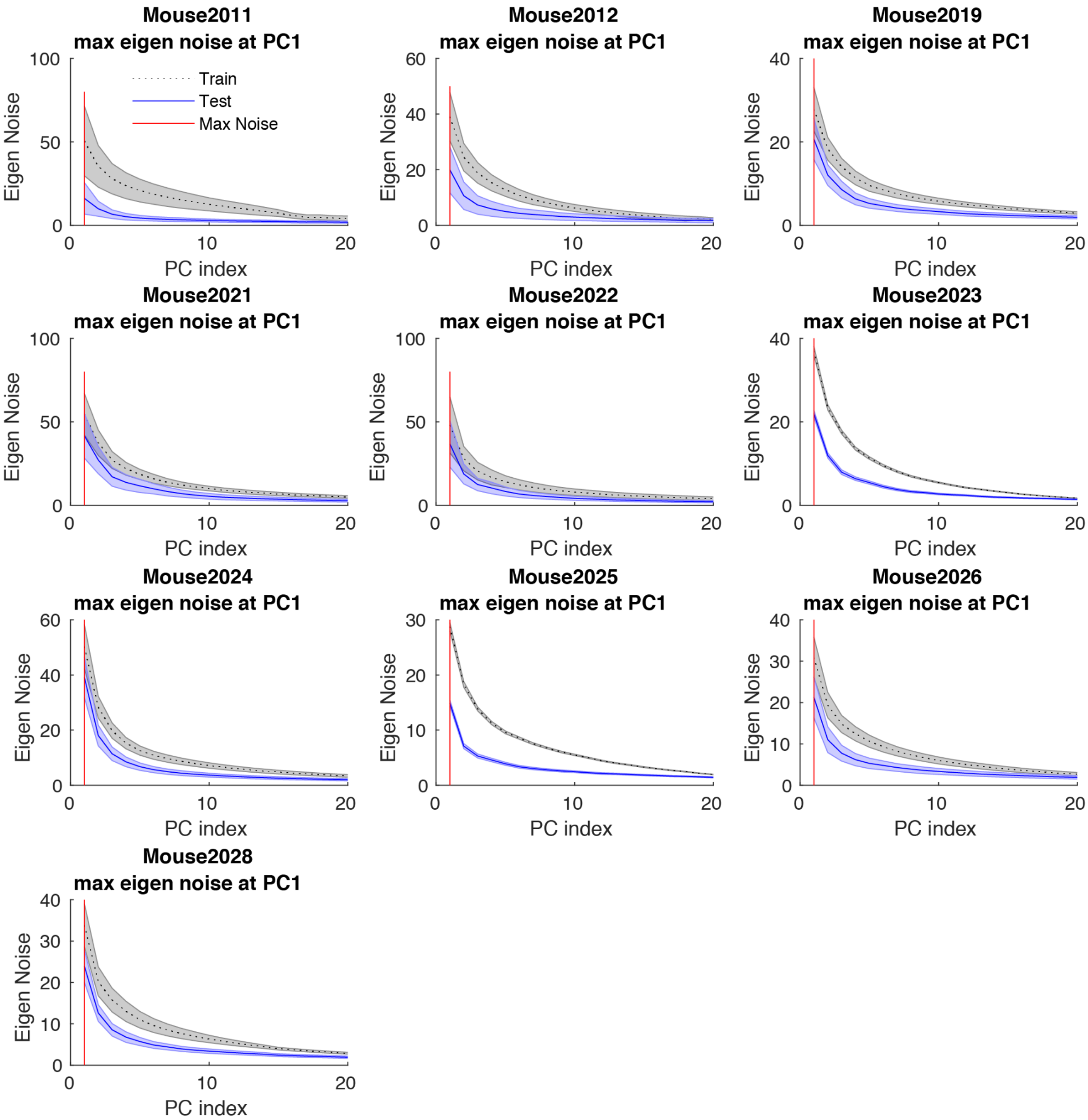

Supplementary Figure 14: The noise along each of the noise principal component directions. The noise along each of the noise covariance matrix eigenvectors is shown separately for each mouse. Showing the noise on the training set (dotted gray) and the testing set (solid blue). We plotted the median noise over spatial bins for each mouse, averaged over 100 train/test sets, and aggregated over the available sessions ($n = 73$ sessions from 10 mice). We denote the dimensions with the highest noise (red line). The shaded regions represent 95% confidence intervals based on the combined SEM across train-test splits and sessions.

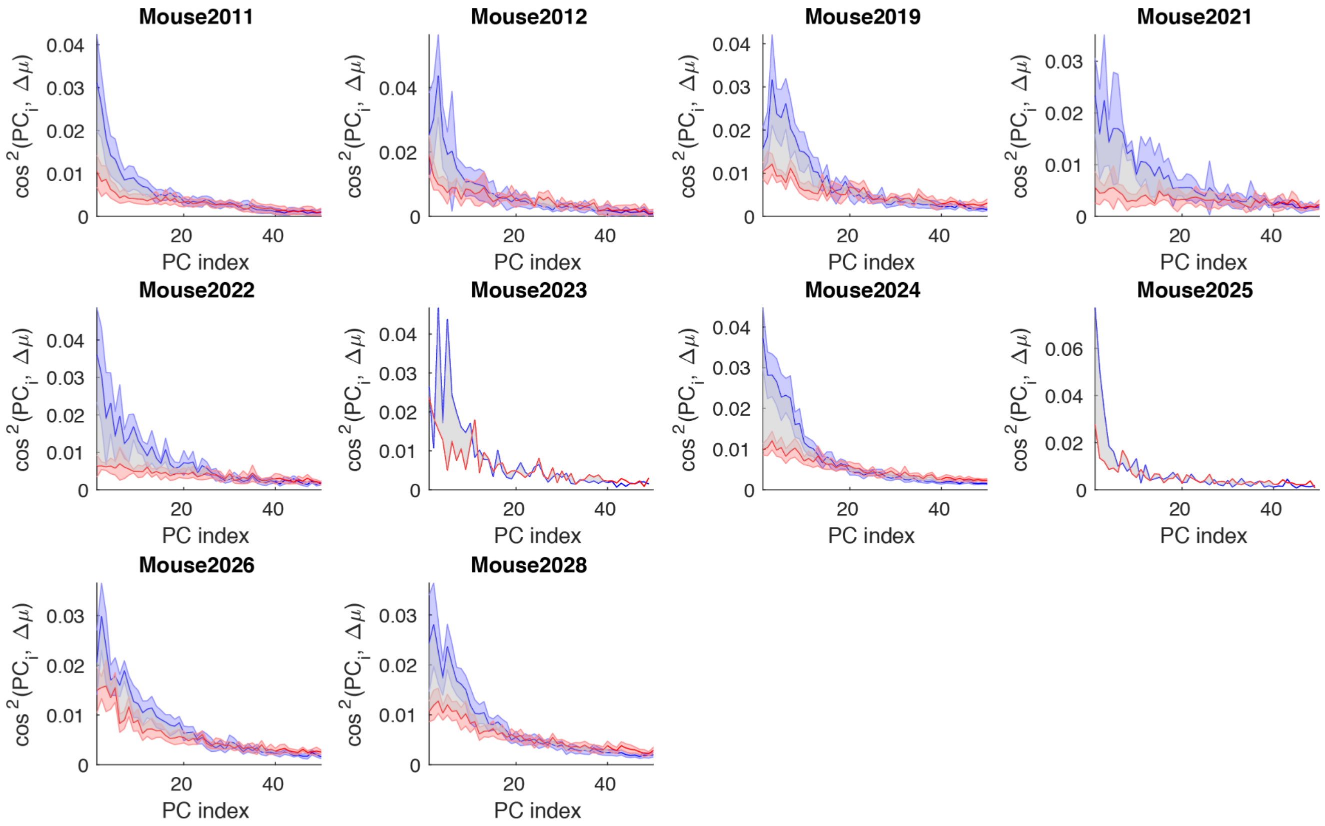

Supplementary Figure 15: The overlap between the signal direction and each noise principal component direction. The squared cosines of the angles between the signal direction $\vec{\Delta\mu}$ and the eigenvectors of the noise covariance matrix (blue) and the shuffled noise covariance matrix (red) are shown separately for each mouse. We used the area between the two curves (gray) to quantify how noise correlations increased the overlap between the noise modes and the signal direction. We plotted the median over spatial bins for each mouse and aggregated over the available sessions (n = 73 sessions from 10 mice). The shaded regions around the curves represent 95% confidence intervals based on the SEM across sessions. These were omitted in mice 2023 and 2025 since each contributed a single session.

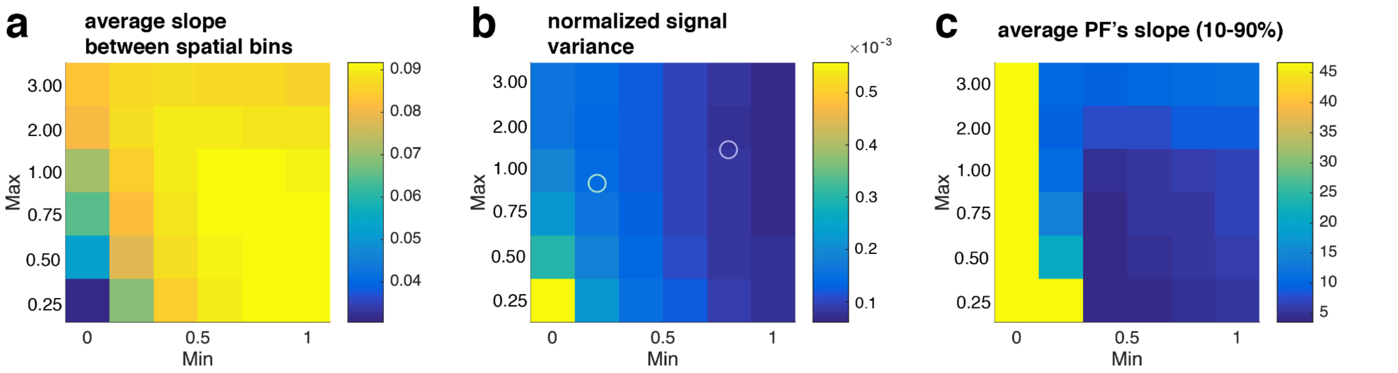

Supplementary Figure 16: Study of how the PF widths distribution changes the PF slopes' values between spatial bins and the shape of the slope distributions measured using the normalized signal variance. The widths distribution is generated using a uniform distribution of widths with two parameters: the minimum and maximum of the uniform range of values. **a)** A heat map of the relation between the parameters of the halfwidth distribution and the mean slope between two adjacent spatial bins (Note: The values are averaged over bins and over 1000 realizations of 250 neurons each). **b)** A heat map of the relation between the parameters of the halfwidth distribution and the mean normalized signal variance. Two circles mark the parameter values for the two distributions used in Figure 5a-d. **c)** A heat map of the relation between the parameters of the widths distribution and the [10-90]% slope of the entire place field (as in Suppl. Fig. 2e).

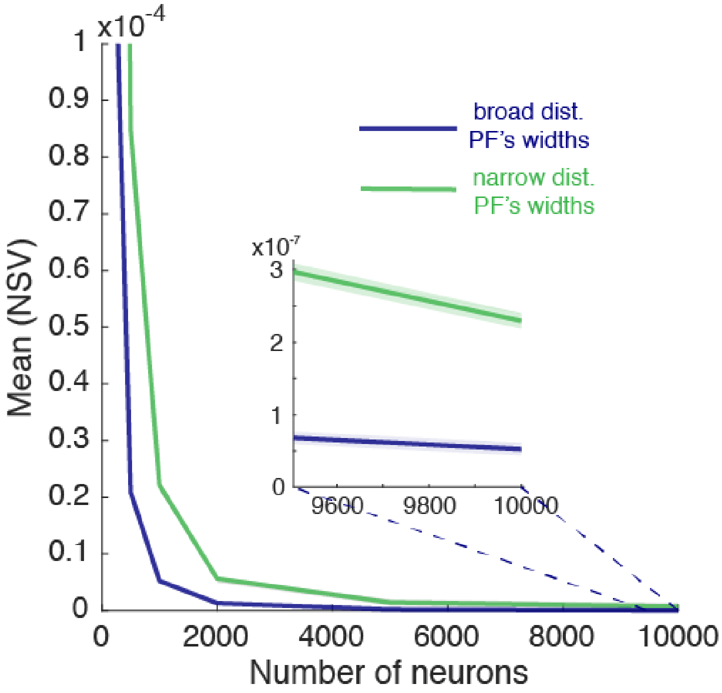

Supplementary Figure 17: NSV computed for the synthetic population of gaussian shapes PFs for different ensemble sizes. The narrow distribution of PFs' width has a higher mean value of NSVs (green line) than a broad width distribution of PFs (blue line) for ensemble sizes up to ten thousand neurons. **Inset:** NSV for the two different distributions of PFs' widths (narrow vs. broad). Solid lines are the average over 500 realizations. The shaded area is the 95% conf-interval for 500 realizations at each ensemble size.

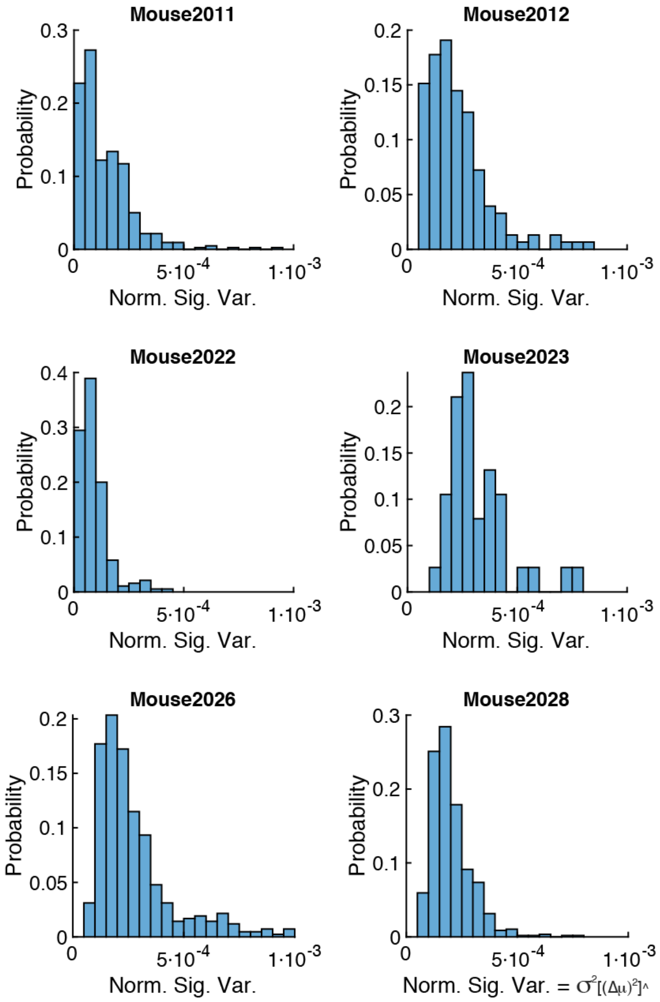

Supplementary Figure 18: The distributions of normalized signal variance (NSV) across spatial bins and sessions. The NSV (see Methods) values across all spatial bins, combined across all available sessions, are shown separately for each mouse (n = 73 sessions from 10 mice). The NSV values vary across a broad range within mice but appear to have distinct central values across mice.

Supplementary Table 2: Correlations between the asymptotic SNR ratio to various predictor variables.

Correlation to (asymp. SNR shuf. / asymp. SNR), across 73 sessions > 200 neurons	Pearson ρ	p-value	Adjusted R^2	Pearson ρ (aggregated)	p-value (aggregated)	Adjusted R^2 (aggregated) = Variance explained
$Var_i((\hat{\Delta\mu}_i)^2)$ = NSV	0.76	1.6×10^{-14}	0.57	0.83	0.0029	0.65
$\sum_i^K \cos^2(\hat{PC}_{corr,i}, \hat{\Delta\mu}) - \cos^2(\hat{PC}_{shuf;corr,i}, \hat{\Delta\mu})$	-0.42	0.00027	0.16	-0.74	0.015	0.48
$Var_i((\Delta\mu_i)^2)$	0.24	0.041	0.044	0.68	0.029	0.40
# neurons	-0.54	9.3×10^{-7}	0.28	-0.61	0.059	0.30
$\frac{(\sum_i^K \lambda_i)^2}{\sum_i^K \lambda_i^2}$	-0.27	0.021	0.060	-0.58	0.079	0.25
$Mean_i((\Delta\mu_i)^2)$	0.13	0.27	0.0033	0.56	0.090	0.23
$\sum_i^K \cos^2(\hat{PC}_{cov,i}, \hat{\Delta\mu}) - \cos^2(\hat{PC}_{shuf;cov,i}, \hat{\Delta\mu})$	-0.37	0.0013	0.13	-0.54	0.11	0.20
$Var_i((PF's\ halfwidths_i))$	-0.21	0.082	0.029	-0.51	0.13	0.17
$\frac{(\sum_i^K \cos^2(\hat{PC}_{cov,i}, \hat{\Delta\mu}))^2}{\sum_i^K \cos^4(\hat{PC}_{cov,i}, \hat{\Delta\mu})}$	-0.13	0.27	0.0036	-0.49	0.15	0.15
# trials	-0.18	0.12	0.020	-0.49	0.15	0.15
$\frac{(\sum_i^K \cos^2(\hat{PC}_{corr,i}, \hat{\Delta\mu}))^2}{\sum_i^K \cos^4(\hat{PC}_{corr,i}, \hat{\Delta\mu})}$	0.013	0.91	-0.014	-0.35	0.32	0.013

We used ten explanatory variables to test for a significant correlation to the effect of correlations on the asymptotic SNR in the position encoding direction. We used 73 sessions from 10 mice with over 200 neurons and excluded data points wherein the confidence interval was larger than the estimated value. Aggregated means average within mice. The rows are sorted based on the adjusted R^2 values. Whenever applicable, quantities are compared over adjacent bin pairs within each session. The variables are:

1. $Var_i((\hat{\Delta\mu}_i)^2)$: Variance of squared elements in the signal direction unit vector (NSV)
2. $\sum_i^K \cos^2(\hat{PC}_{corr,i}, \hat{\Delta\mu}) - \cos^2(\hat{PC}_{shuf;corr,i}, \hat{\Delta\mu})$: The difference in area between the real and shuffled curves of the cosine squared of the angle between the signal direction and each noise correlations eigenvector (that was possible to compute given data constraints).

3. $Var_i((\Delta\mu_i)^2)$: The variance of squared elements in the signal vector (unnormalized).
4. The number of neurons in each session.
5. $\frac{(\sum_i^K \lambda_i)^2}{\sum_i^K \lambda_i^2}$: The effective number of noise modes found using PCA on the covariance matrix.
6. $Mean_i((\Delta\mu_i)^2)$: The mean of the squared elements in the signal vector (unnormalized).
7. $\sum_i^K \cos^2(\hat{PC}_{cov,i}, \hat{\Delta\mu}) - \cos^2(\hat{PC}_{shuf;cov,i}, \hat{\Delta\mu})$: The difference in area between the real and shuffled curves of the cosine squared of the angle between the signal direction and each noise covariance eigenvector (that was possible to compute given data constraints).
8. $Var_i((PF's\ halfwidths_i))$: The variance over the place-fields half-widths within a session
9. $\frac{(\sum_i^K \cos^2(\hat{PC}_{cov,i}, \hat{\Delta\mu}))^2}{\sum_i^K \cos^4(\hat{PC}_{cov,i}, \hat{\Delta\mu})}$: The inverse sum of fourth powers of the signal direction vector in the covariance PCA eigenbasis.
10. The number of trials in the session.
11. $\frac{(\sum_i^K \cos^2(\hat{PC}_{corr,i}, \hat{\Delta\mu}))^2}{\sum_i^K \cos^4(\hat{PC}_{corr,i}, \hat{\Delta\mu})}$: The inverse sum of fourth powers of the signal direction vector in the correlations PCA eigenbasis.

<u>Symbol definitions:</u>

1. $\Delta\mu_i$: The difference in the mean population vector between adjacent spatial bins, indexed at neuron *i*.
2. σ_i: The pooled standard deviation across two adjacent spatial bins for neuron *i*.
3. $\hat{x}_i$: The vector *x*, normalized and indexed at neuron *i*.
4. *K*: The maximal PCA mode index that was possible to compute given data constraints.
5. $\cos^2(a, b)$: The squared cosine of the angle between vectors *a* and *b*.
6. $\hat{PC}_{corr,i}$ or $\hat{PC}_{cov,i}$: The PCA eigenvector for index *i*, in the context of correlation or covariance PCA, respectively. An additional subscript of *shuf* indicates that the vector comes from shuffled data.
7. λ_i: The eigenvalue from covariance PCA with index *i*.

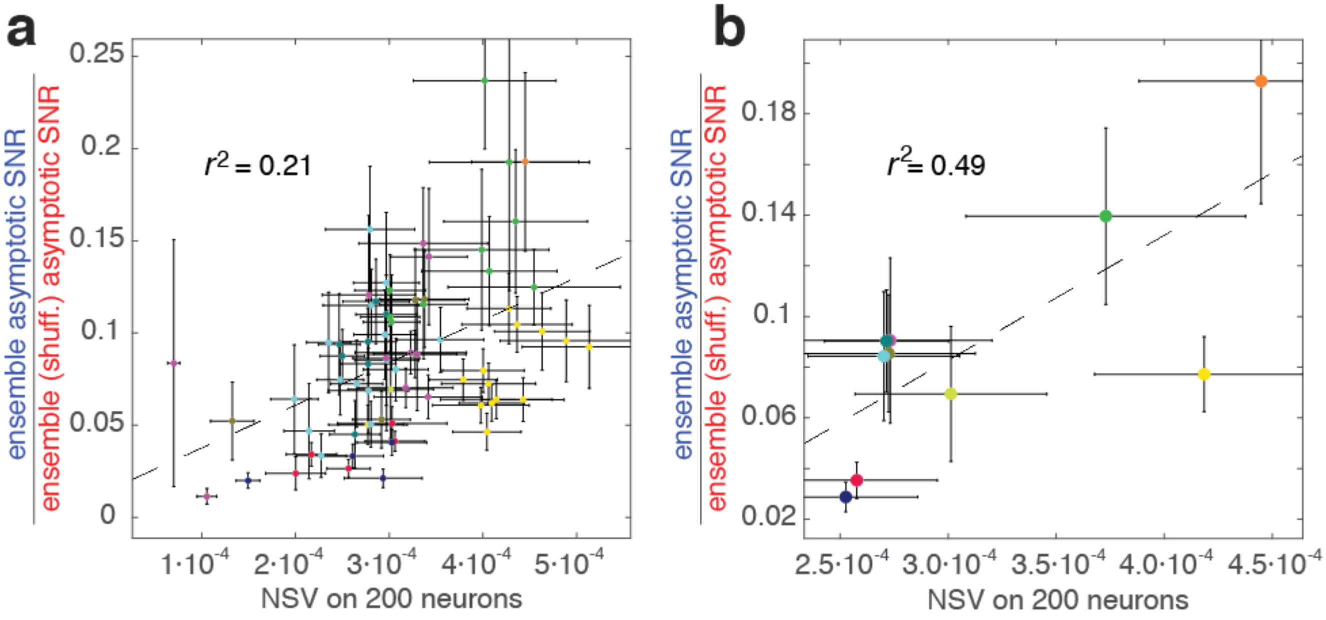

Supplementary Figure 19: Control for ensemble size to explain NSV correlation with asymptotic SNR ratio. We have found that the ensemble size does not explain the correlation between the asymptotic ratio and NSV. The ratio between the asymptotic SNR for un-modified data versus correlation free data (i.e., the asymptotic ratio) correlates with the ensemble size (Suppl. Table 2). This is because the larger the ensemble, the farther apart the two asymptotic SNR curves, the smaller the ratio value. To test if the number of neurons plays a role in the correlation between the asymptotic ratio and NSV, we fixed the number of neurons for all the analyzed sessions and corroborated that the correlation holds. **a)** correlation between the asymptotic ratio and NSV is significant for each session. Pearson: 0.470, $p = 3x10^{-5}$. Color-coded animal names (see Suppl. Fig. 11a). Each dot corresponds to the average over different neurons' subsets for a given session. Error bars are the 95% confidence interval. **b)** correlation between the asymptotic ratio and NSV is significant among animals: Pearson: 0.738, p = 0.01; Spearman: 0.636, p = 0.05; Kendall: 0.556, p = 0.03. Mean values and 95% confidence interval as in **a**.

Supplementary References:

1. Cabral, H.O., Fouquet, C., Rondi-Reig, L., Pennartz, C.M. and Battaglia, F.P., 2014. Single-trial properties of place cells in control and CA1 NMDA receptor subunit 1-KO mice. *Journal of Neuroscience*, 34(48), pp.15861-15869.
2. Chen, T.W., Wardill, T.J., Sun, Y., Pulver, S.R., Renninger, S.L., Baohan, A., Schreiter, E.R., Kerr, R.A., Orger, M.B., Jayaraman, V. and Looger, L.L., 2013. Ultrasensitive fluorescent proteins for imaging neuronal activity. Nature, 499(7458), pp.295-300.
3. Frank, L.M., Brown, E.N. and Wilson, M., 2000. Trajectory encoding in the hippocampus and entorhinal cortex. Neuron, 27(1), pp.169-178.
4. Kelemen, E. and Fenton, A.A., 2010. Dynamic grouping of hippocampal neural activity during cognitive control of two spatial frames. PLoS biology, 8(6), p.e1000403.
5. Lee, I., Rao, G. and Knierim, J.J., 2004. A double dissociation between hippocampal subfields: differential time course of CA3 and CA1 place cells for processing changed environments. Neuron, 42(5), pp.803-815.
6. Mizuseki, K., Diba, K., Pastalkova, E., Teeters, J., Sirota, A. and Buzsáki, G., 2014. Neurosharing: large-scale data sets (spike, LFP) recorded from the hippocampal-entorhinal system in behaving rats. *F1000Research*, *3*.
   Reference for data source: Mizuseki, K., Sirota, A., Pastalkova, E., Diba, K., Buzsáki, G. (2013) Multiple single unit recordings from different rat hippocampal entorhinal regions while the animals were performing multiple behavioral tasks. CRCNS.org.
   http://dx.doi.org/10.6080/K09G5JRZ
7. Raveau, M., Polygalov, D., Boehringer, R., Amano, K., Yamakawa, K., and McHugh, T.J., 2018. Alterations of in vivo CA1 network activity in Dp (16) 1Yey Down syndrome model mice. Elife, 7, p.e31543.
8. Stringer, C., Michaelos, M., Tsyboulski, D., Lindo, S.E., and Pachitariu, M., 2021. High-precision coding in visual cortex. *Cell*, *184*(10), pp.2767-2778.
