# Supplementary material for: Noise correlations in neural ensemble activity limit the accuracy of hippocampal spatial representations
Source: Nat Commun. 2022 Jul 25;13:4276. doi: 10.1038/s41467-022-31254-y (PMC9314334; doi:10.1038/s41467-022-31254-y)
Supplement: Supplementary file 2 — Reporting Summary [file 41467_2022_31254_MOESM2_ESM.pdf]

nature research

Corresponding author(s): Pablo E. Jercog
Last updated by author(s): May 20, 2022

Reporting Summary

Nature Research wishes to improve the reproducibility of the work that we publish. This form provides structure for consistency and transparency in reporting. For further information on Nature Research policies, see our Editorial Policies and the Editorial Policy Checklist.

Statistics

For all statistical analyses, confirm that the following items are present in the figure legend, table legend, main text, or Methods section.

n/a	Confirmed	
☐	☒	The exact sample size (*n*) for each experimental group/condition, given as a discrete number and unit of measurement
☐	☒	A statement on whether measurements were taken from distinct samples or whether the same sample was measured repeatedly
☐	☒	The statistical test(s) used AND whether they are one- or two-sided<br>*Only common tests should be described solely by name; describe more complex techniques in the Methods section.*
☐	☒	A description of all covariates tested
☒	☐	A description of any assumptions or corrections, such as tests of normality and adjustment for multiple comparisons
☐	☒	A full description of the statistical parameters including central tendency (e.g. means) or other basic estimates (e.g. regression coefficient) AND variation (e.g. standard deviation) or associated estimates of uncertainty (e.g. confidence intervals)
☐	☒	For null hypothesis testing, the test statistic (e.g. *F*, *t*, *r*) with confidence intervals, effect sizes, degrees of freedom and *P* value noted<br>*Give P values as exact values whenever suitable.*
☒	☐	For Bayesian analysis, information on the choice of priors and Markov chain Monte Carlo settings
☒	☐	For hierarchical and complex designs, identification of the appropriate level for tests and full reporting of outcomes
☐	☒	Estimates of effect sizes (e.g. Cohen's *d*, Pearson's *r*), indicating how they were calculated

Our web collection on statistics for biologists contains articles on many of the points above.

Software and code

Policy information about availability of computer code

Data collection	We collected the data utilizing nVista2 mini-microscopes
Data analysis	We analyzed the calcium data using MATLAB R2018b and custom code which can be found at github.com/approbatory/ML-project. We also used the CellMAX algorithm which is available through the calcium-Imaging Analysis package (github.com/bahanonu/calciumImagingAnalysis). We used the NoRMCorre piecewise linear registration algorithm (Pnevmatikakis et al. 2017) for calcium data pre-processing. We used OpenCV2 v.3.1.0 to analyze the position of the animals from he videos synchronized with the mini-microscopes. The cameras where controlled using IC-capture 2.4 software. The identification of the animal's center of mass in the video was performed using BackgroundSubtractorMOG2 library from OpenCV.

For manuscripts utilizing custom algorithms or software that are central to the research but not yet described in published literature, software must be made available to editors and reviewers. We strongly encourage code deposition in a community repository (e.g. GitHub). See the Nature Research guidelines for submitting code & software for further information.

Data

Policy information about availability of data

All manuscripts must include a data availability statement. This statement should provide the following information, where applicable:
- Accession codes, unique identifiers, or web links for publicly available datasets
- A list of figures that have associated raw data
- A description of any restrictions on data availability

We provide a link to the public repository "http://crcns.org/data-sets/hc" to download all the data used in the analysis of the results presented in the manuscript. The data will be sufficient to reproduce all the results. No extra data will be provided by any further request from readers.
We will also provide a excel file with all the points to reproduce all figures on the manuscript.

April 2020

Field-specific reporting

Please select the one below that is the best fit for your research. If you are not sure, read the appropriate sections before making your selection.

- [x] Life sciences
- [ ] Behavioural & social sciences
- [ ] Ecological, evolutionary & environmental sciences

For a reference copy of the document with all sections, see nature.com/documents/nr-reporting-summary-flat.pdf

Life sciences study design

All studies must disclose on these points even when the disclosure is negative.

Sample size	We used 12 mice to show that any measured phenomenon is not specific to one individual, and to be able to assess correlations between measured observables across subjects. The sample size was not calculated based on animal's groups statistical result, since results are obtained for each individual sessions from each individual animal and then replicated in all 12. Animals where the recorded neurons were less than 150 neurons, were not used in this work.
Data exclusions	We excluded experimental sessions in which we identified fewer than 30 instances of stereotyped running behaviors or fewer than 150 measured neurons in the calcium imaging data. We performed the exclusion in order to provide enough information to the computational decoders to be able to estimate the decoding accuracy in the limit of a large neuronal ensemble. For some analyses we further excluded sessions with fewer than 200 neurons in order to tighten the estimated confidence intervals. We pre-established the exclusion of sessions based on the number of running behaviors and detected neurons but did not pre-establish the threshold numbers themselves.
Replication	The experiment presented in this manuscript can be considered as an independent test for each of the 110 sessions obtainment from several sessions for each of the 12 animals. All results presented in this work were replicated in all 110 sessions taken as independent experiments.
Randomization	The mice were not divided into different experimental groups.
Blinding	Blinding was not relevant to our study because the study did not divide subjects into different experimental groups.

Reporting for specific materials, systems and methods

We require information from authors about some types of materials, experimental systems and methods used in many studies. Here, indicate whether each material, system or method listed is relevant to your study. If you are not sure if a list item applies to your research, read the appropriate section before selecting a response.

Materials & experimental systems

n/a	Involved in the study
x	Antibodies
x	Eukaryotic cell lines
x	Palaeontology and archaeology
	x Animals and other organisms
x	Human research participants
x	Clinical data
x	Dual use research of concern

Methods

n/a	Involved in the study
x	ChIP-seq
x	Flow cytometry
x	MRI-based neuroimaging

Animals and other organisms

Policy information about studies involving animals; ARRIVE guidelines recommended for reporting animal research

Laboratory animals	Male C57BL/6J (14-16 weeks)
Wild animals	No wild animals were used in the study
Field-collected samples	No field collected samples were used in the study
Ethics oversight	Stanford University Administrative Panel on Laboratory Animal Care

Note that full information on the approval of the study protocol must also be provided in the manuscript.
